# Toward a Predictive Coding Account of Hypnotic Hypoalgesia: Integrating EEG Oscillations and Somatosensory Evoked Responses

**DOI:** 10.3390/brainsci16080779

**Published:** 2026-07-23

**Authors:** Vilfredo De Pascalis

**Affiliations:** 1 Department of Psychology, Sapienza University of Rome, 00185 Rome, Italy; vilfredo.depascalis@fondazione.uniroma1.it; 2 School of Psychology, University of New England, Armidale, NSW 2351, Australia

**Keywords:** hypnotic hypoalgesia, pain modulation, EEG, somatosensory event-related potentials, predictive coding, active inference, theta, alpha, beta, gamma, hypnotizability, clinical hypnosis

## Abstract

**Highlights:**

**What are the main findings?**

- Hypnotic hypoalgesia is clearly linked to cooperative changes across theta, alpha, beta, and gamma oscillations, indicating a multiband modulation rather than a single frequency-specific mechanism.
- Later evaluative components of somatosensory event-related potentials are more consistently affected by pain relief during hypnosis than early sensory responses, suggesting altered salience attribution and affective-cognitive appraisal of pain.

**What are the implications of the main findings?**

- Predictive coding and active inference frameworks explain pain relief via hypnotic analgesia as a shift in the precision, meaning, and threat value of nociceptive signals, rather than as sensory suppression.
- These mechanisms may support more individualized hypnotic interventions by guiding the content of suggestions based on pain type, expectancy, hypnotizability, agency, and treatment goals.

**Abstract:**

Hypnotic hypoalgesia effectively reduces various types of pain, but its neurophysiological mechanisms remain unclear. EEG and somatosensory event-related potentials (SERPs) can help elucidate how hypnotic suggestions influence pain processing across sensory, emotional, and cognitive domains. This review synthesizes evidence on EEG oscillatory activity and SERP correlates in hypnotic pain modulation and interprets the findings through predictive coding and active inference frameworks. The review integrated relevant studies on EEG frequency-band oscillations, SERPs, and the modulation of pain by hypnosis, focusing on a convergent neurophysiological account of hypnotic pain modulation rather than on individual studies. Research indicates that hypnotic hypoalgesia is associated with concerted changes across multiple EEG frequency bands. Theta activity is linked to focused internal attention and the maintenance of suggestion-consistent representations, while increased alpha activity may indicate inhibitory sensory gating and reduced nociceptive gain. Beta oscillations enhance pain relief in cognitive and motor functions, while decreased gamma activity indicates reduced significance of pain signals. SERP findings show that hypnotic suggestions primarily affect late evaluative components (e.g., P200/P250, P300) rather than early sensory elements, suggesting changes in salience assignment and affective-cognitive appraisal rather than uniform suppression of nociceptive input. Predictive coding and active inference explain how hypnotic suggestions change pain-related expectations and bodily perceptions. This insight is key to enhancing personalized pain management and guiding future neuroscience research.

## 1. Introduction

Pain is considered a complex experience, comprising the sensory-discriminative, affective-motivational, and cognitive-evaluative components, which are subserved by distinct yet dynamically interacting neural networks that integrate nociceptive input with attention, expectation, memory, affect, contextual meaning, as well as broader emotional and sociocultural factors [1,2,3].

Since hypnosis can selectively influence different components of pain depending on the content of the verbalized suggestions, pain modulation via hypnosis represents an important method that can potentially be used in patient-centered clinical practice. Beyond its effects on pain perception itself, accumulating evidence suggests that pain-modulating interventions, including hypnotic hypoalgesia, may also influence social-emotional processing and empathy. These findings further support the view that pain modulation extends beyond nociceptive processing to encompass broader affective and interpersonal functions [4]. Suggestions targeting pain unpleasantness modulated activity in the anterior cingulate cortex (ACC) without comparable changes in the somatosensory cortex [5]. In contrast, suggestions targeting pain intensity were associated with modulation of activity in the primary somatosensory cortex [6]. More recent research supports the view that hypnotic suggestions can differentially target the sensory and affective-evaluative components of pain [7]. These findings indicate that hypnotic hypoalgesia is a suggestion-specific process rather than a nonspecific consequence of hypnotic induction alone.

The clinical benefits of hypnotic hypoalgesia are well-established. Hypnosis can reduce acute procedural pain, experimental pain, and several forms of clinical pain, with larger effects generally observed in individuals with medium-to-high hypnotizability [8,9,10,11]. However, how hypnotic suggestions alleviate pain is not fully understood. It is improbable that hypnotic hypoalgesia can be explained solely by distraction, relaxation, or response bias. Current experimental evidence indicates that hypnosis affects multiple levels of pain processing, including oscillatory coordination, attentional control, affective estimation, agency, and the conscious integration of bodily sensations.

Electroencephalography (EEG) is particularly useful for exploring these mechanisms because it provides temporal resolution of about 1 millisecond through various signal analysis methods. In addition, EEG enables the analysis of both real-time oscillatory activity and stimulus-locked evoked responses to noxious stimulation. Signal processing of EEG oscillatory activity typically examines changes in classic frequency-band oscillations, such as theta (4–8 Hz), alpha (8–13 Hz), and gamma (>35 Hz). In addition, somatosensory event-related potentials (SERPs) provide ordered response indices of sensory encoding, motivational significance, and evaluative processing. Together, these methods allow us to investigate whether hypnotic hypoalgesia influences early nociceptive encoding, later cognitive-affective appraisal, or both [12].

Predictive coding (PC) and active inference offer a useful theoretical framework for integrating and explaining EEG/SERP findings. The brain actively constructs plausible hypotheses about incoming sensory information [13]. Recent work has extended this perspective by proposing that hypnotic susceptibility and trait mindfulness share common neural mechanisms, including enhanced theta- and alpha-band oscillatory activity, prefrontal–cingulate interactions, and dynamic modulation of large-scale brain networks within an interoceptive PC framework, suggesting that these phenomena may rely on overlapping computational principles of conscious state regulation [14]. Within this framework, the brain is an active player rather than a passive receiver of sensory inputs. Perception is not a passive encoding of sensory input, but an inferential process in which prior expectations and incoming sensory input are combined in the brain while accounting for uncertainty [15]. This interpretation implies that prediction errors influence perception according to their precision (i.e., their estimated reliability or gain) [16,17,18]. Pain is not viewed as a direct readout of nociceptive input but as an inferential process arising from the integration of sensory signals with prior expectations and changes in precision (i.e., nociceptive input is interpreted in light of expectations, threat assessments, contextual meanings, and prior beliefs about bodily state) [19,20,21]. This mechanistic view indicates that the suggestions of hypoalgesia during hypnosis can modify higher-order generative models. This alteration changes expectations about bodily sensations and affects how incoming pain signals are processed [22,23,24,25,26].

Although several reviews have examined the clinical efficacy of hypnosis for pain or summarized neuroimaging findings associated with hypnosis, no recent review has specifically integrated EEG oscillatory activity and somatosensory event-related potentials (SERPs) within a unified mechanistic framework (for more detailed information, see the Supplementary Materials). The present review seeks to bridge this gap by organizing electrophysiological findings according to their proposed computational roles in pain perception, including attentional allocation, precision weighting, sensory gating, and the evaluation of nociceptive prediction errors. Rather than providing a descriptive catalog of studies, the review develops a mechanistic interpretation linking electrophysiological observations to contemporary computational theories of brain function. This strategy is necessary since the literature contains convergent, dissociated, and mixed findings: some studies show parallel reductions in pain and neurophysiological markers; others show subjective hypoalgesia without early SERP changes; still others indicate that hypnotic hypoalgesia and distraction can produce similar pain reductions through different neural paths (for a literature review, see [12]).

Accordingly, the principal contribution of this narrative review is not the presentation of new experimental data but the development of an integrative conceptual framework. By interpreting EEG oscillations and SERP findings through PC and active inference, the review proposes a coherent account of how hypnotic suggestions may alter the precision, behavioral relevance, and hierarchical processing of nociceptive information. This perspective also identifies unresolved questions and provides testable hypotheses for future electrophysiological investigations of hypnotic pain modulation.

Through this integrative approach, the current review aims to highlight how electrophysiological markers can bridge basic neuroscience with clinical practice. It may inform the mechanisms, efficacy, and optimization of hypnosis-based pain interventions, potentially contributing to the development of more effective, personalized, and evidence-based clinical pain treatments [8,27].

## 2. Methods

This article is a narrative review that synthesizes current evidence on the neurophysiological mechanisms underlying hypnotic hypoalgesia, with particular emphasis on electroencephalographic (EEG) oscillatory activity, somatosensory evoked/event-related potentials (SERPs), and their interpretation within the PC and active inference frameworks.

The literature search was performed using the PubMed, Scopus, and Web of Science databases. The final search was completed in January 2025 and updated through July 2026. Search terms included combinations of the following keywords: hypnosis, pain, hypnotic hypoalgesia, EEG, electroencephalography, EEG band oscillations, EEG band functional connectivity, EEG network connectivity, somatosensory evoked potentials, laser-evoked potentials, somatosensory event-related potentials, predictive coding, and active inference. Boolean operators (AND/OR) were used to combine search terms as appropriate. Additional relevant publications were identified through manual screening of the reference lists of retrieved articles and pertinent review papers.

Eligible publications included peer-reviewed original research articles, clinical studies, systematic and narrative reviews, and theoretical papers addressing the neurophysiology of hypnosis, pain modulation, EEG correlates, SERPs, or PC mechanisms. Preference was given to studies conducted in humans and to articles considered foundational or highly influential in the field. Conference abstracts, duplicate publications, non-peer-reviewed reports, and studies not directly relevant to the scope of this review were excluded.

The retrieved literature was screened by the author for relevance based on titles, abstracts, and where necessary, full texts. As the objective of this work was to provide a critical and integrative overview of the field rather than a systematic review or meta-analysis, a formal assessment of methodological quality or risk of bias was not conducted.

## 3. EEG/MEG Oscillatory Correlates of Hypnotic Hypoalgesia

EEG and magnetoencephalography (MEG) are complementary techniques for recording neural activity with millisecond temporal resolution. EEG measures the electrical activity of cortical pyramidal neurons, whereas MEG records the magnetic fields induced by these currents. The two methods therefore reflect closely related neuronal phenomena but differ in their sensitivity to source orientation and tissue-related signal distortion. EEG is sensitive to both radial and tangential sources but is more affected by volume conduction through the skull and scalp. MEG is especially sensitive to tangential cortical sources, exhibits less spatial smearing, and offers advantages for source localization, although it is more expensive, less widely available, and less portable than EEG [28].

In pain research, EEG and MEG have been especially valuable because pain depends on rapidly changing interactions among sensory, attentional, affective, and contextual processes. Pain-related evoked potentials and oscillatory responses are not determined by a single neural feature but by complex spatial–temporal–spectral patterns of brain activity. This aspect is particularly relevant for hypnotic hypoalgesia, where analgesic suggestions may alter both stimulus-locked responses and ongoing oscillatory activity across theta, alpha, beta, and gamma bands. At present, however, direct MEG evidence specifically examining hypnotic hypoalgesia remains limited compared with EEG, SERP, PET, and fMRI evidence. Future combined EEG–MEG studies could help identify whether hypnotic hypoalgesia primarily modulates early sensory generators, later evaluative networks, or distributed oscillatory interactions involving somatosensory, insular, cingulate, and prefrontal regions [29,30].

Although direct MEG evidence on hypnotic hypoalgesia remains scarce, MEG findings from hypnotic anesthesia are relevant. In a study, MEG was used during vibrotactile stimulation of the left hand under hypnotically induced anesthesia, revealing reduced beta-band activity at 214–413 ms, mainly localized to the right insula, and reduced alpha-band activity at 253–500 ms, mainly localized to the left inferior frontal gyrus. Because a second experiment ruled out attention modulation alone, these findings suggest that hypnotic sensory attenuation may involve top-down inhibitory mechanisms within frontal–insular networks [31].

Although EEG bands should not be interpreted too strictly, they provide useful language for describing how neural systems coordinate perception, attention, memory, affective evaluation, and cognitive activity. Pain-evoked oscillatory responses typically include EEG alpha and beta suppression, increases in theta and gamma, and enhancement in sensorimotor, insular, and cingulate regions [32,33,34]. These responses are not dependent on pain intensity; they are instead influenced by factors such as attention, expectation, arousal, stimulus intensity, task demands, and emotional significance.

Oscillatory changes are often described in terms of event-related synchronization (ERS) or event-related desynchronization (ERD) of oscillatory power across multiple frequency bands [35]. The functional significance of ERS and ERD responses is dependent on the frequency band in which they occur. The occurrence of an ERS response in the alpha frequency band is usually interpreted as an indicator of inhibition or reduced information processing in cortical areas that are not relevant to the task. This response can also be seen as a temporary disengagement of the brain region from active information processing [36]. Conversely, an ERD response in the alpha range indicates a decrease in relative power compared to the baseline, decreased synchrony within local neural networks, and typically reflects increased cortical engagement, reflecting enhanced processing demands from the incoming stimulus [37,38,39]. Importantly, both ERS and ERD capture local, frequency-specific changes in synchrony rather than interactions between distant brain regions [35]. An ERS in the gamma frequency band is frequently linked to stimulus-feature binding, sensory integration, and conscious awareness. However, gamma activity can also be influenced by attention and motor artifacts [34,40,41,42].

Pain modulation through hypnotic suggestions is likely to reflect a reorganization of multiple brain processes involving distinct frequency oscillations, rather than a single gamma-band oscillation. Such modulations may reorganize the balance between slow-wave attentional control, attenuation of ascending nociceptive signals, and fast-wave sensory-evaluative integration.

### 3.1. Theta Oscillations and Hypnotic Responsiveness

Theta activity has been closely linked to factors such as susceptibility to hypnosis, focused attention, imagination, working memory, and creativity. Mark Jensen and his team have advanced an interesting “slow wave hypothesis” according to which higher slow-wave activity (theta, alpha, or both) is significantly associated with enhanced responsiveness to hypnotic suggestions and may serve as a predictor of which individuals will benefit most from hypnotic treatment [43,44]. If this hypothesis is valid, the increased theta activity observed during hypnotic hypoalgesia may reflect the ongoing process of effectively facilitating the delivery of pain-relief suggestions, thereby helping to focus attention on a positive internal experience of reduced discomfort.

One important finding comes from the study by Houzé et al. [45]; among various comparisons between conditions, time-frequency analysis of EEG activity revealed a significant difference between distraction and hypnotic hypoalgesia in the theta domain. Although the authors observed that both pain control methods successfully reduced pain, they found that these procedures influenced theta activity differently: distraction lowered frontal-midline theta, while hypnotic hypoalgesia enhanced it. This finding is important because it shows that hypnotic hypoalgesia is more than just a form of distraction. Hypnotic hypoalgesia requires maintaining a sustained attentional focus on internal experiences and mental imagery models driven by suggestion. In contrast, distraction effectively reduces pain by redirecting attention away from harmful sensory input.

More generally, recent EEG connectivity research indicates that theta-band activity supports directed communication between anterior control regions and posterior representational systems, consistent with a broader role of theta oscillations in coordinating internally guided cognitive processes [46].

Clinical work also supports a role for theta, although the evidence remains preliminary. Mark Jensen et al. [47] examined whether theta-enhancing neurofeedback or mindfulness training could improve subsequent self-hypnosis treatment in patients with multiple sclerosis and chronic pain, fatigue, or both. Left-anterior theta neurofeedback was associated with larger improvements in pain, sleep disturbance, pain interference, and depression following hypnosis treatment. In contrast, mindfulness meditation was associated with larger improvements in pain acceptance. These findings suggest that theta enhancement may facilitate subsequent response to self-hypnosis treatment. However, this conclusion remains preliminary because the study involved a small sample (*n* = 32) and a clinically heterogeneous group of participants. EEG-guided personalization of hypnotic interventions is therefore promising, but it should be regarded as an experimental approach requiring further validation before routine clinical application.

Other evidence complicates the theta hypothesis. In a study using virtual-reality hypnosis, researchers reported decreased delta and increased theta activity at the onset of painful electrical pulses [48]. These observations highlight an interesting trend across the two low-frequency EEG bands, likely influenced by the hypnotic induction method and the specific approach used to deliver painful stimuli. Importantly, other studies have shown no consistent increase in theta activity during hypnosis, even among highly hypnotizable individuals [49,50]. In some reviews, it is therefore emphasized that theta is not a simple biomarker of hypnotizability or hypnotic condition [51,52]. A reasonable conclusion is that enhanced theta activity plays a significant role in the induction of hypnotic hypoalgesia and in sustaining analgesic expectations, especially when suggestions involve mental imagery that requires focused internal attention. However, it is important to note that changes in theta are heavily influenced by context, the nature of the suggestions, and the analytical methods employed.

Within a PC conceptualization, perception relies on the relative precision assigned to descending predictions and ascending sensory evidence. During hypnosis, suggestions for pain relief may enhance the effect of prior expectations about bodily sensations and reduce the influence of pain signals on the conscious experience of pain [16,17,22,23,24,26]. Accordingly, suggestions that the stimulated body part is numb, distant, protected, or less threatening may establish an alternative generative model of bodily sensation. Thus, the rise in theta activity during hypnotic hypoalgesia can be seen as a direct reflection of the implementation process of suggestion-driven priors, rather than merely indicating a suppression of prediction error. Theta activity may contribute to this process since theta enhancement has been linked to sustained hypnotic attentional engagement, maintenance of suggestion-consistent representations, and working memory, functions that are central to both hypnotic responding and the top-down modulation of pain [43,44,45,53,54]. Theta activity should be considered a marker of attention and an electrophysiological measure of prediction error. This caution is necessary because findings on theta waves during hypnosis vary across studies and can depend on factors such as hypnotizability, task demands, induction procedure, type of suggestion, and contextual factors [44,51,52].

### 3.2. Alpha Oscillations and Inhibitory Sensory Gating

Original research shows that increased alpha activity may help reduce pain perception [55]. This association is likely due to increased alpha-band oscillations (8–12 Hz), which may reflect inhibitory sensory gating of irrelevant stimulation, thereby making more capacity available for processing relevant information [56,57]. Thus, a notable rise in alpha activity during hypnotic hypoalgesia provides strong support for the idea that alpha-band oscillations play a vital role in gating relevant information by functionally inhibiting irrelevant (undesired, painful) sensory information [58].

Some studies report increases in alpha activity, while others report decreases or no change during hypnotic hypoalgesia. This finding appears to depend on hypnotizability levels, the specific suggestions used, and recording sites [59]. Along these lines, a study on placebo hypoalgesia treatment in waking and hypnosis conditions reported that during waking, the enhancement of relative left-parietal alpha2 (10–12 Hz) power directly influenced pain reduction, and indirectly through the mediating positive effect of subjective involuntariness. Instead, the enhancement of left temporoparietal alpha2 power to placebo hypoalgesia in hypnosis influenced pain reduction only through the mediation of subjective involuntariness [60]. This distinction is theoretically important because it suggests that placebo hypoalgesia during waking and hypnosis involves different processes of top-down regulation and reinforces the need to examine hypnosis and its effects on pain perception via neurophysiological mechanisms. This line of research can help us implement effective pain management techniques that significantly improve the quality of life for people in pain.

In a study on pain relief using virtual-reality hypnosis, the authors reported a reduction in perceived pain intensity, along with increased EEG power in the 5–11 Hz range, suggesting modulation of activity spanning theta and lower-alpha frequencies [48].

Thalamocortical circuits play a central role in synchronizing neural oscillations and regulating the transmission of sensory information [61,62,63]. Changes in the synchronization of these circuits may therefore provide an important framework for understanding how hypnosis influences attention, perception, and conscious awareness. Within this context, hypnotic hypoalgesia has been hypothesized to depend not only on reductions in perceived pain intensity, but also on changes in how pain is consciously perceived and experienced [64]. The idea that hypnotic hypoalgesia may originate from an inhibitory mechanism is consistent with the concept that increased low-frequency brain activity, particularly in the theta and alpha bands, can enhance an individual’s susceptibility to hypnosis [43,65] and improve responsiveness to hypnotic suggestions [44].

Because alpha oscillations have been linked to functional inhibition, cortical gating, and reduced sensitivity of task-irrelevant sensory channels [56,57,58,66], the PC interpretation of alpha-band activity is relatively clear but should be expressed cautiously. Alpha enhancement may indicate a decrease in sensory gain or a lower level of precision allocated to nociceptive input [16,67,68]. In other words, incoming pain signals may still be present, but their effective influence on higher-level inference is reduced. In the domain of pain, alpha activity has been linked to decreased pain perception and sensory inhibition. This view supports the idea that enhancing alpha-band activity may lead to hypoalgesia by reducing the effects of incoming nociceptive signals [48,55,60,69]. Nevertheless, alpha activity is not specific to hypnosis or hypoalgesia; it may indicate reduced sensory gain, attentional inhibition, cortical idling, or other processes depending on the context [36,56,57,59,70,71,72]. Consequently, alpha activity should be viewed as a component of a larger multiband and network-level pattern, rather than being considered an autonomous indicator of hypnotic pain control [43,44,52,73].

### 3.3. Beta Oscillations and Cognitive-Sensorimotor Set

Although beta band activity (13–35 Hz) has received less attention in hypnotic analgesia research than theta, alpha, and gamma bands, clinical research findings outside hypnosis suggest an important role for beta oscillations, particularly in clinical populations. In addition, beta rhythms are crucial for understanding pain modulation because they are associated with sensorimotor integration, cognitive control, and the maintenance of current perceptual or motor tasks [74,75,76]. Beta activity suppression is commonly observed following noxious stimulation, particularly in sensorimotor regions, suggesting enhanced sensory processing or a change in the sensorimotor state [33,77,78].

In hypnosis, beta modulation may indicate the maintenance or reconfiguration of suggestion-driven bodily expectations. A hypoalgesic suggestion requires participants to maintain a modified interpretation of their bodies and their body’s sensations over time. This modification may involve changes in beta-band activity associated with the maintenance of a new cognitive-sensorimotor set. Original studies on hypnotizability indicated increased activity in the high-theta, high-alpha, and beta bands among individuals with high hypnotizability during emotional imagery in hypnosis, especially in the right parietal regions [79]. In the same research line, EEG studies suggest that hypnosis leads to changes in frontal functional organization and connectivity. These changes include patterns associated with hypofrontality, dissociated control, and altered executive monitoring [80,81,82]. Such research findings indicate that beta activity may play a role in sustaining attentional focus, imagery-related tasks, and control processes during hypnosis, especially when suggestions involve maintaining an altered sense of body awareness.

Recent clinical EEG findings provide additional support for a multiband interpretation of the hypnotic modulation of pain. In a high-density EEG study, Kumar et al. [83] assessed hypnosis-related resting-state EEG activity in patients with fibromyalgia and chronic pain undergoing hypnosis. These authors observed that during the hypnosis condition (suggestion to relive a pleasant autobiographical memory), there was an increase in theta power in the left parietal and occipital sites, an increase in beta power in the frontal and left temporal sites, and an increase in slow-gamma power in the frontal and left parietal sites. These findings underscore that hypnosis in chronic pain may reorganize large-scale multiband activity even when immediate pain relief is not evident.

From a PC perspective, beta oscillations may reflect the stabilization of a current generative model or cognitive set. Beta activity is associated with maintaining the current sensorimotor or cognitive state and with exerting top-down control over ongoing processing [67,74,75,76,84]. Accordingly, beta modulation during hypnotic hypoalgesia may indicate the maintenance of analgesic expectations or the reorganization of prefrontal control mechanisms that support suggestion-driven bodily perceptions [22,25,26,80,81,82,85]. However, this interpretation remains indirect, as beta-band mechanisms have been tested less systematically in hypnotic hypoalgesia compared to late ERP components or effects in the theta, alpha, and gamma bands. Beta activity should therefore be included in the theoretical conceptualization. Still, it should not be given the same evidential weight as the more extensively studied electrophysiological markers of hypnotic pain modulation [12,44,52,86].

### 3.4. Gamma-Band Dynamics, Pain, and Conscious Integration

Although gamma band oscillations (35–100 Hz) have generally been regarded as a marker of fine-grained neural integration [87,88] and sensory feature binding (the binding-by-synchronization hypothesis) [89], current EEG research on the neural correlates of nociceptive pain has well-established the essential role of high-frequency EEG activity in the gamma band in pain processing [90].

Multiple EEG studies have shown a strong relationship between EEG gamma activity and perceived pain intensity, e.g., see [32,91,92], as well as stimulus strength, particularly in the sensorimotor cortex and pain-related networks [32,34,91,92,93]. These findings highlight gamma oscillations as a key indicator of pain processing. They are particularly relevant to hypnotic hypoalgesia, as effective analgesic suggestions may diminish the integration of nociceptive signals into the conscious experience of pain.

Gamma activity has been shown to predict subjective pain ratings in a non-hypnotic control condition, but this relationship was no longer present during hypnosis in highly hypnotizable participants [94]. In a related study, phase-ordered gamma responses to painful stimulation were positively associated with subjective pain ratings during waking, whereas this association disappeared during hypnotic hypoalgesia. Gamma responses were also reduced during hypnotic hypoalgesia, particularly among high- and medium-hypnotizable participants [95]. These findings indicate that hypnosis may disassociate gamma-band activity from the experience of pain, thereby changing the typical relationship between nociceptive processing and perceived pain.

Hypnotic hyperalgesia provides a complementary perspective. Suggestions of hyperalgesia and hypoalgesia have been shown to modulate subjective pain intensity and unpleasantness in highly hypnotizable participants [96]. Hyperalgesic instructions increased perceived pain and were associated with enhanced P200 amplitudes and gamma-band synchronization in response to nociceptive laser stimulation. This opposite pattern supports the view that hypnosis can either increase or decrease the evaluative weight assigned to pain-related input, depending on the content of the suggestion.

In hierarchical PC views, gamma activity has been associated with bottom-up communication during sensory processing and local cortical sensory integration; meanwhile, beta synchronization affords feedback communication, refining top-down predictions for a more dynamic understanding of sensory experiences [67,84,97,98]. However, theoretical work also cautions against interpreting gamma oscillations as specific carriers of prediction errors [67,84,98,99,100]. This interpretation is crucial for understanding pain, as research has demonstrated a strong link between gamma-band activity and nociceptive processing and self-reported pain intensity. Furthermore, studies on pain modulation during hypnosis have shown that in cases of hypnotic hypoalgesia, gamma activity decreases, and there is a disconnection between gamma responses and pain ratings [32,34,91,92,94,95,96]. However, this interpretation requires caution. Gamma activity is also strongly influenced by selective attention, arousal, task demands, motor activity, microsaccades, muscle artifacts, and analytic procedures; therefore, it cannot be treated as a specific marker of prediction error [40,101,102,103,104,105]. Thus, gamma reductions or gamma–pain decoupling should be taken as evidence that hypnosis primarily alters evaluative processing, rather than as direct proof that nociceptive prediction errors have been suppressed.

### 3.5. Multiband and Network Dynamics

The most reliable conclusion from the oscillatory literature is that hypnotic hypoalgesia arises from a complex interplay among multiple electromagnetic oscillations in the brain, rather than from a single frequency [12].

Enhanced theta activity could support a strong focus on internal thoughts, improving working memory, and helping maintain imaginative suggestions during hypnosis and pain management [44,45,106,107].

Alpha activity could play a key role in reducing sensory input, i.e., filtering out distracting stimuli and managing pain signals, as well as in functional inhibition and reduced nociceptive gain or precision, thereby limiting the impact of task-irrelevant or pain-related sensory input on higher-level processing [56,57].

Beta activity may play a crucial role in refining the ongoing cognitive-sensorimotor set, which stabilizes bodily expectations in alignment with suggestions during hypnosis [75,76,83,84].

Gamma activity may indicate the significance, conscious awareness, and sensory-evaluative integration of pain-related signals. However, this interpretation should be approached with caution, as gamma activity is also influenced by attention, focused arousal, and analytical processes [32,34,40,91,92,94,95,96].

These multiple frequency oscillatory bands involved in pain and its modulation do not operate independently. Pain perception and hypnotic modulation depend on distributed interactions among somatosensory cortices, insula, anterior cingulate cortex, prefrontal regions, thalamocortical loops, and descending pain-modulatory systems [1,2,3,33,108]. The MEG findings from hypnotic anesthesia further support the view that hypnosis-related somatosensory attenuation may involve coordinated alpha–beta modulation within frontal–insular networks rather than a single frequency-specific mechanism [31].

Cross-frequency coupling presents a promising yet underexplored approach for understanding how these oscillatory processes might interact. Theta–gamma coupling aligns predictions with sensory experiences, while alpha–gamma and alpha–beta interactions promote sensory inhibition and enhance top-down sensory control [58,109,110,111,112,113].

While direct evidence linking cross-frequency coupling to reduced pain during hypnosis is currently limited, this manuscript highlights it as a significant and promising area for future research, and has the potential to open the way for a deeper understanding of pain management through hypnosis [12,43,52,114].

The oscillatory findings reviewed above describe how hypnosis may reorganize ongoing brain states, event-related frequency-band responses, and network-level activity. SERPs provide complementary information by indexing time-locked stages of nociceptive processing, from early sensory encoding to later attentional and evaluative appraisal. For this reason, the next section examines SERPs as an additional electrophysiological window onto hypnotic pain modulation.

## 4. Somatosensory Event-Related Potentials and Hypnotic Modulation of Pain

Research shows that early components of somatosensory event-related potentials (SERPs) reflect the initial processing of nociceptive input and are associated with activity in somatosensory and operculo-insular systems involved in pain encoding [21,115,116]. In contrast, later SERP components, such as P200/P250, N2, and P300, are linked to attentional allocation, the significance of nociceptive input, expectancy, context updating, and affective evaluation [115,116,117,118]. The P200/P250 is a positive deflection occurring approximately 200–250 ms after stimulation and is often associated with early evaluative processing. The N2 is a negative deflection occurring around 200–350 ms and is commonly related to salience detection, conflict, or stimulus evaluation. The P300 is a later positive component, typically occurring between 300 and 600 ms, and is associated with attention, context updating, and conscious evaluation of stimulus significance.

Across studies, hypnotic hypoalgesia more consistently modulates late than early SERP components. SERP studies demonstrate that hypnotic suggestions of hypoalgesia and hyperalgesia can respectively increase or decrease SERP responses and the behavioral relevance of nociceptive stimulation [96,119,120,121]. Reductions in P200/P250, P300, and vertex-complex amplitudes are associated with subjective pain relief, suggesting reduced processing of nociceptive information [48,119,120,122,123,124,125]. However, other studies show that subjective analgesia can occur without significant changes in early sensory event-related potential (SERP) components [45,126,127,128,129,130], suggesting pain perception can be altered even when early nociceptive encoding is preserved.

Conversely, studies indicate that effective hypnotic suggestions in highly hypnotizable individuals can reduce both early and late components of the spontaneous electrical activity of the reticular activating system (SERP) [48,121,131,132]. Such findings are compatible with stronger top-down modulation of nociceptive precision and sensory gain. Cases in which pain relief occurs without clear SERP changes [45,126,127,128,129,130] suggest that additional mechanisms—such as oscillatory network dynamics, interoceptive inference, autonomic regulation, agency-related processes, and metacognitive assessment—may also contribute to hypnotic analgesia [26,133].

In conclusion, experimental evidence suggests that hypnotic hypoalgesia primarily influences our perception and interpretation of pain signals, rather than merely blocking sensory transmission [5,6,22,26].

Taken together, SERP findings suggest that hypnotic hypoalgesia does not necessarily depend on reduced early electrophysiological responses to nociceptive input. Instead, pain relief often appears to involve later stages of processing, including attention, affective evaluation, and the assignment of motivational significance to pain-related stimuli. This pattern provides the rationale for interpreting electrophysiological evidence within predictive coding and active inference frameworks.

## 5. Predictive Coding and Active Inference Accounts

PC models propose that perception emerges from reciprocal interactions between top-down predictions and bottom-up prediction errors, with higher levels of the cortical hierarchy generating predictions about the causes of sensory input and lower levels transmitting mismatch signals when incoming evidence deviates from those predictions [15,17,68,134]. According to a PC view, the brain continuously estimates the hidden causes of sensory input and updates its perceptual hypotheses whenever new evidence is discrepant with its previous expectations [15,17,135]. Precision assignment determines how prediction errors influence perception by modulating their impact on sensory inference. High-precision prediction errors drive the updating of internal models, which in turn facilitate learning and adaptation. In contrast, when prior expectations are given greater precision (such as expectations about pain relief from hypnotic suggestion), they have a stronger impact on sensory interpretation. This influence can change how incoming pain signals are perceived, ultimately reducing the intensity or significance of the pain [16,17,136].

Pain is well-suited for PC analysis because nociceptive input is influenced by expectation, threat, attention, emotion, prior learning, and context [21,137]. Placebo analgesia, nocebo hyperalgesia, chronic pain, and fear learning are representative phenomena demonstrating that pain is a multifaceted experience that extends well beyond the mere intensity of nociceptive signals [19,20,21,108]. For example, it is clearly accepted that hypnotic hypoalgesia operates through inferential mechanisms, induced by the explicit use of suggestions to create alternative bodily experiences. These experiences clearly involve sensations of numbness, distance, and protection, as well as a notably reduced sense of threat from the painful body part [5,6,22,26,138]. Recent theoretical developments also support the view that PC may involve more distributed higher-order cognitive operations than originally proposed, emphasizing the role of prefrontal mechanisms in generating and evaluating prediction errors rather than restricting these computations to early sensory hierarchies [139].

Computationally, three frameworks are particularly relevant to understanding the brain mechanisms underlying hypnotic suggestions and the modulation of pain by hypnosis (for more details, see Section S2 in the Supplementary Materials).

**Figure 1:**
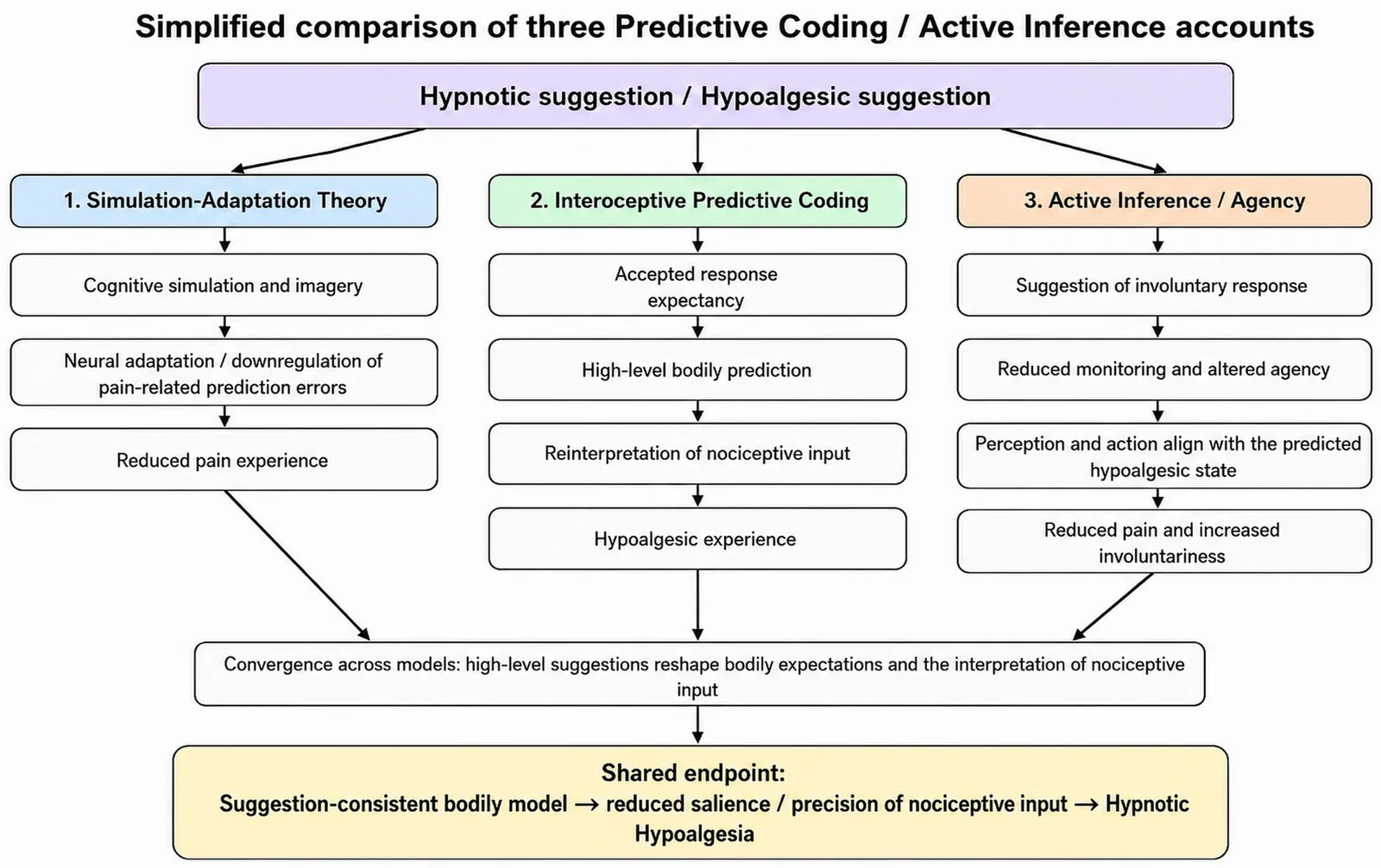
Simplified comparison of three predictive-coding and active-inference accounts of hypnotic hypoalgesia. The figure summarizes three complementary models through which hypnotic suggestions may reduce pain. The simulationadaptation theory emphasizes cognitive simulation, mental imagery, and neural adaptation to representations, consistent with suggestions [23]. The interoceptive predictive-coding account emphasizes response expectancy, high-level bodily predictions, and reinterpretation of nociceptive input [22,24]. The active-inference/agency account emphasizes altered agency, reduced voluntary monitoring, and alignment of perception with the predicted hypoalgesic state [26]. Although these accounts differ in emphasis, they converge on a shared endpoint: hypnotic suggestions may establish a suggestion-consistent bodily model that reduces the salience or precision of nociceptive input, thereby contributing to hypnotic hypoalgesia.

To improve accessibility for readers without a computational background, the three accounts can be understood as complementary rather than competing models. They differ mainly in the mechanisms emphasized: cognitive simulation and neural adaptation; interoceptive expectancy and bodily prediction; or altered agency and active inference. However, all three converge on the idea that hypnotic suggestions modify high-level expectations about bodily state and thereby change how nociceptive input is interpreted (see Figure 1).

The first framework is the simulation-adaptation theory of hypnosis, which posits that hypnotic analgesia arises from sustained cognitive simulation and focused attention on pain representations that align with the suggested directives. This process results in an adaptive downregulation of the precision of pain-related prediction errors [23].

The second view explains phenomena of hypnotic suggestions by emphasizing the primary importance of the participant’s acceptance of response expectancies. According to this perspective, hypnotic suggestions are most effective when incorporated into higher-order predictions about bodily state and interoceptive experience [22,24,25].

The third account focuses on active inference and highlights the concepts of agency and involuntariness. According to this view, effective hypnotic responses are perceived as involuntary due to strong expectations. This powerful influence reduces the need for voluntary monitoring or executive correction, leading to a smoother, more enjoyable experience [26].

The insula and cingulate cortex play central roles in PC, active inference, and current models of hypnotic hypoalgesia. Activity in the posterior insula has been linked to sensory-interoceptive representation and awareness of bodily states, including pain [140,141,142]. In contrast, the anterior insula integrates bodily signals with salience, cognitive control, and emotion processes [141,143]. The ACC plays a central role in the motivational and emotional aspects of pain, in monitoring conflicts and the significance of nociceptive input, and in regulating control [5,144,145]. Together, the insula and cingulate systems contribute to the detection and prediction of bodily threats, as well as to the updating of pain-related beliefs and expectations [21,146]. In chronic pain, persistent nociceptive prediction errors and maladaptive precision assignment are thought to contribute to pain persistence, even when peripheral nociceptive input is reduced or ambiguous [137,147,148]. Effective hypnotic suggestions may recalibrate these inferential processes by reducing threat-related expectations, enhancing perceived safety, and reshaping the interpretation of bodily sensations [5,22,26,138].

Active inference extends PC by emphasizing action. In this framework, prediction errors can be minimized not only by adjusting beliefs, but also by altering the environment to make sensory input or body perception more consistent with prior predictions [149,150]. In the context of pain, protective actions and avoidance or withdrawal can be seen as our body’s way of safeguarding itself and working toward restoring its well-being [151,152]. In chronic pain, however, protective behaviors may become maladaptive if they reinforce threat expectations and maintain pain-related avoidance. Hypnotic analgesia may therefore operate by changing both perception and action tendencies, e.g., the body part may be experienced as numb, safe, distant, or protected, and the patient may reduce defensive monitoring or protective actions [26,153].

This framework emphasizes the significant role of subjective involuntariness in hypnotic responding, both in practice and theory. Involuntariness, as the fundamental suggestion effect of hypnotic responding, is directly linked to shifts in agency, metacognitive awareness, and distinct control experiences [26,154,155]. When patients feel that an analgesic response is occurring involuntarily, they tend to trust the suggested bodily state more. For example, the suggestion “your hand is becoming numb” can be more powerful when the numbness happens spontaneously, rather than through conscious effort. This interpretation aligns with findings that during hypnotic placebo hypoalgesia, faster alpha (alpha2) activity plays a key role in reducing pain by influencing our perceptions of control [60].

## 6. Integrated Evidence Patterns

Having reviewed EEG oscillations, SERP findings, and predictive-coding interpretations separately, this section integrates these lines of evidence into recurring empirical patterns. This pattern-based organization is intended to show how apparently heterogeneous findings can be understood within a common framework while recognizing that hypnotic hypoalgesia may involve multiple interacting processes, including attentional control, sensory gating, affective evaluation, altered salience, and precision modulation.

1. Several studies demonstrate a consistent late-evaluative response pattern that is sensitive to pain modulation during hypnosis, in which a reduction in subjective pain is associated with decreases in the late components of the event-related potential (ERP), specifically the P200/P250, P300, and vertex-complex responses. These observations support the hypothesis of reduced motivational significance and diminished evaluative processing of nociceptive information [48,119,120,122,123,124,125]. Computationally, these findings suggest diminished salience-weighted nociceptive prediction errors and a heightened role for analgesic expectations in the conscious experience of pain [16,17,21].
2. Some studies report reductions in early components such as N100/N140, P150, or earlier sensory-related responses (e.g., N20), especially in highly hypnotizable participants or under strong analgesic suggestions [48,120,121,131,132]. However, early SERP modulation is inconsistent across studies and is not considered a defining feature of hypnotic hypoalgesia here.
3. A pattern is characterized by the dissociation between subjective hypoalgesia and relatively preserved early sensory responses. Numerous studies unequivocally show that pain reduction can occur even in the absence of significant changes in early- or mid-latency SERP components [45,126,127,128,129,130]. These observations indicate that higher-order processes such as reinterpretation, expectancy, interoceptive inference, agency, and metacognition can fundamentally alter the pain experience without requiring significant suppression of nociceptive encoding [22,26,133].
4. Another set of studies has shown that although both hypnotic hypoalgesia and distraction reduce pain, these suggestions do not necessarily produce similar EEG or SERP changes consequent to pain reduction [45,129,156].
5. Another pattern concerns the relationship between EEG oscillatory activity and pain-control mechanisms. Changes in EEG bands—theta, alpha, beta, and gamma—distinguish hypnotic hypoalgesia from other forms of pain modulation, such as distraction. Enhanced theta activity clearly indicates a strong connection between pain relief, sustained attention, and the effective maintenance of represented expectations [45,47,48]. Enhanced alpha synchronization may reflect suppression of sensory input, significant inhibitory control, and pain reduction, mediated by subjective involuntariness [60]. Beta activity is found to stabilize cognitive-sensorimotor sets, while gamma activity is observed to be fundamentally tied to sensory relevance and the bottom-up integration of sensory evaluations [32,34,56,57,58,75,76,91].
6. A recurring finding is the reduction or disappearance of the association between gamma activity and subjective pain reports during hypnotic hypoalgesia, suggesting altered integration of nociceptive signals into conscious awareness [94,95]. However, gamma activity should be interpreted cautiously because it is influenced by attention, arousal, motor activity, and analytical procedures [34,40,91,102].
7. Studies comparing hypnotic hypoalgesia and hyperalgesia show that hypnotic suggestions can either reduce or amplify the perception and subjective significance of painful stimuli, all depending on the delivered specific suggestion [96,119,120,121].
8. Hypnotic hypoalgesia may be paralleled by changes in autonomic markers, i.e., heart rate variability (HRV), heart rate (HR), skin conductance response (SCR), and subjective measures such as involuntariness, dissociation, or virtual reality-induced absorption, suggesting that pain modulation involves interoceptive and agency-related processes as well as sensory processing [45,48,157,158].
9. Finally, clinical pain studies demonstrate robust evidence that hypnosis and related interventions can significantly influence theta, alpha, beta, and slow-gamma brain activity. From clinical pain studies, it can be derived that chronic pain is primarily driven by altered network dynamics, rather than by a single oscillatory abnormality. In addition, the interplay of pain and hypnosis involves interactions among multiple oscillatory bands. Specific interactions, such as theta–gamma, alpha–gamma, and alpha–beta, are highly relevant. However, there remains a need for more direct evidence regarding hypnotic hypoalgesia [47,83].

These patterns are summarized in Table 1. The table is organized by evidence pattern rather than by individual study because this better reflects the state of the literature. Individual studies vary in sample size, hypnotizability selection, stimulation method, type of suggestion, ERP/EEG measure, and analytic approach. A pattern-based organization allows convergent, dissociated, and mixed results to be interpreted without forcing all findings into a single mechanism.

The evidence indicates that hypnotic hypoalgesia should be viewed as a complex modulation of pain processing, rather than merely a reduction in nociceptive input. This modulation appears to involve several factors:

(1) Changes in late evaluative processing, which are evidenced by alterations in the components of late auditory event-related potentials (SERPs/ERPs) associated with the detection of stimulus relevance, affective valuation, and context updating.
(2) Oscillatory precision control, shown through changes in theta, alpha, beta, and gamma brain wave bands that may help regulate attention, enhance sensory perception, and integrate pain-related signals.
(3) Regulation of autonomic and interoceptive processes.
(4) A diminished connection between nociceptive input and the conscious experience of pain.

Collectively, these findings suggest that hypnotic hypoalgesia is best understood as a multidimensional modulatory process involving sensory, attentional, affective, and evaluative processes, rather than merely a simple suppression of nociceptive input [12,16,22,133].

## 7. Clinical Implications

A predictive-coding perspective offers a unifying framework for psychopathology and several clinically relevant implications for the application of hypnosis in pain management.

The clinical implications discussed below differ in their level of evidential support. Direct experimental evidence supports the analgesic effects of hypnosis, the influence of suggestion content on sensory versus affective-evaluative pain processing, and the relatively consistent modulation of late rather than early SERP components. Evidence for specific EEG-band mechanisms is suggestive but inconsistent across studies. In contrast, interpretations related to precision reweighting, active inference, altered agency, and reduced defensive monitoring are theoretically grounded but lack direct validation through computational or experimental tests. Likewise, EEG-guided personalization, neurofeedback, virtual-reality-assisted hypnosis, and combined hypnosis–mindfulness approaches should currently be regarded as preliminary clinical possibilities rather than established interventions. Accordingly, the following implications are presented with explicit distinction between conclusions supported by direct experimental evidence and those that remain theoretically informed or preliminary.

(1) Available experimental comparisons indicate that hypnotic hypoalgesia is not fully explained by distraction, although the number of direct comparative EEG and SERP studies remains limited. While distraction primarily reduces pain by directing attention away from nociceptive input [159,160,161], hypnotic suggestions can also modify the meaning, significance, and interpretation of bodily sensations by reshaping expectations and bodily perceptions [5,6,22,23,26,138].
(2) The content of hypnotic suggestions appears clinically relevant. Neuroimaging findings indicate that suggestions targeting pain intensity and unpleasantness may engage partially distinct neural mechanisms involving somatosensory and cingulate systems [5,6]. These findings provide experimental support for the hypothesis that specific suggestions elicit neural effects. However, the proposal that hypnotic interventions should be tailored to the sensory, emotional, cognitive, and motivational dimensions of an individual patient’s pain remains a clinically plausible hypothesis that requires prospective evaluation [21,108,137,148,162].
(3) Agency, expectancy, and involuntariness are potential therapeutic factors that are supported by both behavioral and clinical observations; however, their causal role in hypnotic analgesia is not yet fully established. From an active-inference perspective, strong expectations of hypoalgesia are understood as high-level predictions about bodily states. These expectations can reduce the need for conscious control and enable suggested responses to be experienced as automatic or involuntary. In this view, altered experiences of agency reflect the successful implementation of predictions that are consistent with suggestions, guide perception and behavior, and minimize prediction error [26,149,150]. Active-inference accounts further predict that effective hypnotic responding may decrease defensive monitoring and maladaptive protective behavior while increasing perceived safety, control, and self-efficacy [26,149,150,151,152]. These proposed mechanisms may be especially relevant to chronic pain, but they have not yet been directly tested as mediators of hypnotic analgesia [21,137].
(4) The proposal that hypnosis and mindfulness are complementary rather than competing interventions is theoretically plausible. However, this notion has not been confirmed by direct comparative studies or by trials combining the two treatments. Mindfulness-based approaches can enhance the awareness of how we think about and interpret pain, including our expectations and habitual reactions to it [163,164]. In contrast, hypnosis can promote a range of different bodily sensations associated with safety, numbness, detachment, coolness, protection, or better control [22,26,47]. Whether combining these approaches produces additive or synergistic clinical benefits remains to be determined.
(5) Preliminary studies suggest that EEG measures, neurofeedback, and virtual-reality-assisted hypnosis may help enhance hypnotic responsiveness or pain relief. However, the available evidence is limited and heterogeneous, and does not currently support their routine use as independent clinical predictors or established therapeutic methods [12,44,47,48,52,165].

Future research should integrate neurophysiological measures with assessments of expectancy, hypnotizability, pain phenotype, emotional distress, and functional outcomes to develop more personalized and effective hypnosis-based interventions [9,138,166,167].

Thus, the strongest clinical conclusions concern the capacity of hypnosis to reduce pain and produce suggestion-specific changes in pain processing. In contrast, the proposed computational mechanisms, biomarker-guided personalization, and combined-treatment applications remain hypotheses requiring prospective validation.

**Figure 2:**
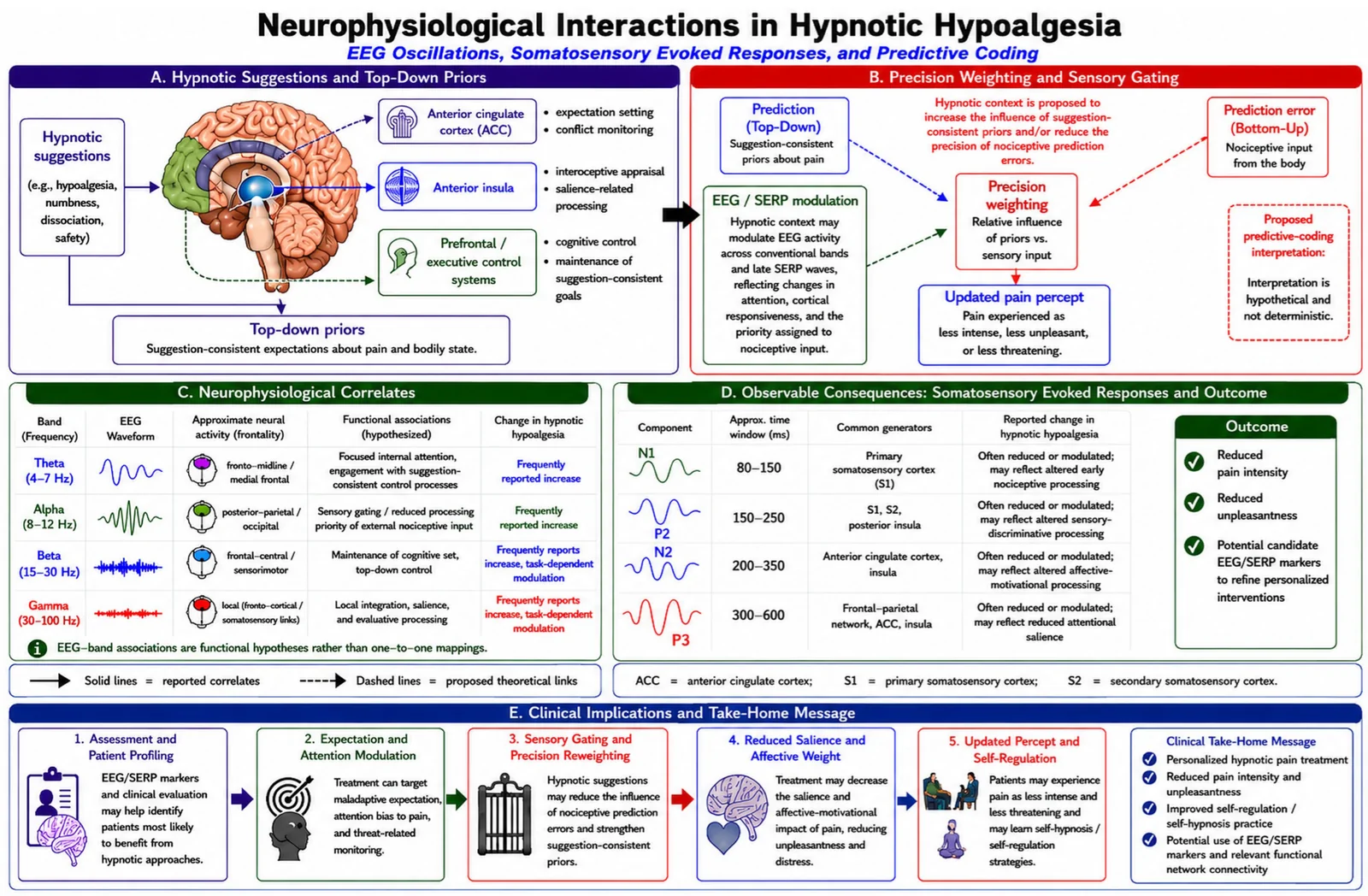
Neurophysiological interactions underlying hypnotic hypoalgesia. The figure integrates predictive coding mechanisms, EEG oscillations, somatosensory evoked responses, and their clinical implications. (**A**) Hypnotic suggestions engage prefrontal/executive systems, the anterior cingulate cortex, and anterior insula, supporting suggestion-consistent expectations and top-down pain-related priors. (**B**) Hypnotic context may alter EEG activity and later somatosensory-evoked response components by altering attention, cortical responsiveness, and the relative precision of top-down predictions and bottom-up nociceptive prediction errors. (**C**) Theta, alpha, beta, and gamma activities are shown with their hypothesized functional associations, approximate cortical distributions, and commonly reported modulation during hypnotic hypoalgesia. (**D**) Representative N1, P2, N2, and P3 components are presented with approximate latency ranges, likely generators, and reported changes. (**E**) Potential clinical applications include patient profiling, expectancy and attention modulation, sensory gating, precision reweighting, reduced pain salience, and enhanced self-regulation. Solid arrows indicate reported empirical associations, whereas dashed arrows indicate proposed theoretical links. ACC, anterior cingulate cortex; EEG, electroencephalography; SERP, somatosensory event-related potential; S1, primary somatosensory cortex; S2, secondary somatosensory cortex.

The proposed integration of EEG oscillatory activity, SERP modulation, predictive-coding mechanisms, and their potential clinical implications is summarized in Figure 2.

### Limitations and Prospective Research Directions

Here are some points, including the main points, summarizing the limitations of the current review and outlining the prospective direction for experimental clinical research.

(1) A general limitation of this narrative review lies in the methodological heterogeneity of the studies examined. The generalizability of the current PC interpretative account is limited by small sample sizes, inconsistent measures of hypnotizability, varying procedures, different types of pain tested, and diverse methods for EEG preprocessing and analysis, e.g., see [43,44,52]. Some studies do not clearly distinguish between the effects of hypnotic induction and those of analgesic suggestion, making it difficult to determine whether observed changes reflect hypnosis per se, relaxation, absorption, expectancy, response motivation, or the specific content of the suggestion, e.g., see [168,169,170,171,172,173]. Furthermore, relatively few hypnosis studies have employed advanced EEG signal analysis methods such as source localization, functional network connectivity, and cross-frequency couplings, e.g., see [43,52,73,174].
(2) PC and active inference explanations were limited because they were derived as theoretically informed interpretations of the reported empirical findings rather than as direct computational tests. It is advisable that in future clinical research, researchers test predictive-coding hypotheses directly from recorded data rather than treating them only as post hoc interpretations. First, studies should more clearly distinguish the effects of hypnotic induction from those of suggestion. In addition, in future studies, a clear distinction between the direction and content of suggestions is essential, as predictive-coding accounts predict that their direction and content should determine whether pain-related priors are strengthened, weakened, or reinterpreted [5,6,172]. Clinical trials should therefore compare suggestions targeting pain intensity, unpleasantness, threat, safety, agency, and bodily ownership.
(3) By combining assessments of, e.g., subjective pain and unpleasantness, absorptive ability and other contextual measures, such as expectancy, individual pain sensitivity, emotional distress, with measures of SERP amplitude and latency, time–frequency analysis of ERS/ERS, source localization, autonomic indices, and computational modeling, future studies should investigate how hypnotic hypoalgesia to phasic pain affects the accuracy of pain-related predictions. For instances: (i) amplitude reductions in late SERP components may be used as an index of altered evaluation of pain and evaluative updating; (ii) enhanced EEG theta and alpha activity consequent to hypnotic hypoalgesia can be used to indicate, respectively, improved attention and enhanced sensory filtering; (iii) a gamma–pain decoupling can indicate less integration of pain signals into conscious experience [45,60,94,95,120]. The use of a formal PC computational modeling approach can disclose whether clinical improvement stems mainly from reduced sensory precision, heightened prior precision, altered interoceptive inference, changes in agency, or more than one of these factors.
(4) Research should examine whether mindfulness and hypnosis can be combined sequentially. Based on the previously mentioned studies [47,163,164], one testable clinical hypothesis is that mindfulness-based monitoring may increase metacognitive awareness of prior pain-related predictions. At the same time, hypnotic treatment may introduce higher-precision alternative priors that support active inference and bodily reinterpretation. Following a possible protocol, patients would first be trained to voluntarily evaluate their pain-related sensations and threat assessments via mindfulness or neurofeedback, and then use suggestions during hypnosis to construct safer bodily models, such as numbness, protection, distance, or confidence in movement. Such a protocol would be especially relevant for chronic pain patients with high threat expectations and maladaptive avoidance.
(5) Clinical trials must adhere to rigorous methodological guidelines. It is important to employ computational Bayesian modeling to estimate prediction errors precisely. Future research must involve adequate sample sizes, standardized hypnotizability assessments, preregistered hypotheses, active control groups, and longitudinal follow-ups. In chronic pain research, it is essential to determine whether suggestive treatment during hypnosis leads to long-lasting changes in patient beliefs about pain, confidence in movement, autonomic function, and activities of daily living, rather than simply providing temporary pain relief. Studies should integrate subjective pain ratings with EEG data, SERPs, autonomic markers, and fMRI, and conduct parallel source- and network-level analyses of EEG. For example, hypotheses derived from a PC modeling framework should be examined by manipulating expectations, precision, uncertainty, and the content of suggestions, and then assessing the effects on pain-relief duration and quality of life.

## 8. Conclusions

This review represents a conceptual contribution by synthesizing diverse electrophysiological findings within a unified computational framework. Rather than treating EEG oscillations and SERPs as separate neurophysiological markers, it interprets hypnotic hypoalgesia within predictive coding and active inference frameworks, suggesting that coordinated changes in brain oscillations and event-related responses may reflect dynamic modulation of perceptual inference, attentional control, and precision assignment across hierarchical neural systems.

Contrary to the oversimplified idea that hypnotic suggestion completely blocks initial pain signals, the evidence demonstrates that hypnotic hypoalgesia primarily affects later evaluative components of SERPs and the dynamic relationship between brain activity and our subjective experience of pain.

Research indicates that EEG theta and alpha activity support, respectively, sustained internal attention and inhibitory control, while beta activity helps maintain hypoalgesic cognitive sets. Gamma activity is associated with the conscious integration of pain signals. Late SERP waves, such as P200/P250, P300, and the vertex complex, are more effectively influenced by pain relief during hypnosis than earlier sensory waves.

It is concluded that hypnotic analgesia can effectively recalibrate the balance between expectation-driven suggestions and nociceptive signals. This neurophysiological conceptualization is consistent with recent clinical evidence indicating that the analgesic benefits of hypnosis are accompanied by changes in pain-related cognitions, including reduced catastrophizing and greater pain acceptance, emphasizing that neural and psychological mechanisms likely operate in concert to produce therapeutic benefit [175]. A better understanding of these interacting mechanisms may help improve personalized hypnosis-based pain management interventions.

## Figures and Tables

**Table 1:** Summarized EEG and SERP evidence for hypnotic modulation of pain interpreted within a predictive-coding and active-inference framework. Evidence is organized by recurrent empirical pattern rather than by individual study. This format emphasizes that Hypnotic Hypoalgesia does not produce a single uniform electrophysiological signature. Instead, the literature shows partially convergent patterns: reductions in late evaluative ERP components, conditional modulation of early sensory components, subjective analgesia without measurable changes in SERPs, theta/alpha-related slow-wave control, gamma–pain decoupling, and agency/interoceptive modulation. The tags classify each pattern as convergent, dissociated, or mixed and identify the likely processing level involved. These tags are interpretive aids rather than judgments of study quality.

Evidence Pattern	Studies Included in the Template	Empirical Synthesis	Predictive Coding/Active Inference Interpretation	Tag	Confidence
1. Late SERP/ERP attenuation during Hypnotic Hypoalgesia	Zachariae & Bjerring (1994) [122]; Crawford et al. (1998) [124]; Danziger et al. (1998) [123]; De Pascalis et al. (2001, 2015) [120,125]; Ray et al. (2002) [119]; Rousseaux et al. (2022) [48]	Hypnotic Hypoalgesia is often associated with reductions in later pain-related components, especially P200/P250, N2, P300, and the vertex complex. These effects are generally stronger in high hypnotizable individuals and are more reliable than reductions in early sensory components.	Reduced late components are consistent with diminished salience attribution, reduced affective-evaluative appraisal, and lower precision assigned to nociceptive signals during belief updating.	C-L	High
2. Conditional early sensory attenuation	Arendt-Nielsen et al. (1990) [121]; De Pascalis et al. (2008, 2015) [120,131]; Perri et al. (2019) [132]; Rousseaux et al. (2022) [48]	Some studies report reductions in early components such as N100/N140, P150, or early sensory-related responses, especially in high hypnotizable participants or under strong analgesic suggestions. However, early modulation is not consistent across studies.	Early reductions may reflect decreased precision weighting of ascending nociceptive prediction errors. Because these effects are conditional, they should not be treated as defining features of Hypnotic Hypoalgesia.	M-E	Moderate
3. Subjective Hypoalgesia without SERP reduction	Amadeo & Yanovski (1975) [126]; Miltner et al. (1993) [128]; Meier et al. (1993) [127]; Friederich et al. (2001) [129]; Williams et al. (2010) [130]; Houzé et al. (2021) [45]	Several studies report significant subjective pain reduction during Hypnotic Hypoalgesia without parallel reductions in early or global SERP amplitudes.	This dissociation suggests that Hypnotic Hypoalgesia can occur through higher-level reinterpretation, reduced salience, or altered appraisal even when early sensory prediction-error signaling remains relatively intact.	D-E/L	High
4. Hypnosis and distraction produce different physiological routes to pain reduction	Schuler et al. (1996) [156]; Friederich et al. (2001) [129]; Houzé et al. (2021) [45]	Hypnosis and distraction can both reduce pain, but they do not necessarily produce the same EEG/SERP profile. Houzé et al. reported theta decreases during distraction but theta increases during Hypnotic Hypoalgesia.	Distraction may reduce pain by reallocating attention away from nociceptive input, whereas hypnosis may stabilize suggestion-driven priors and alter precision weighting within pain inference.	M-O	Moderate–High
5. Theta/alpha slow-wave control pattern	Jensen et al. (2018) [47]; Houzé et al. (2021) [45]; De Pascalis et al. (2021) [60]; Rousseaux et al. (2022) [48]	Theta and alpha activity are frequently implicated in hypnotic responsiveness and hypnotic pain modulation. Theta may support sustained internal attention and maintenance of analgesic suggestions; alpha may support inhibitory sensory gating. Left parietal alpha2 are predicting pain reduction in waking placebo hypoalgesia, while left temporoparietal alpha2 does so in hypnosis through subjective involuntariness.	Theta/alpha changes are consistent with top-down precision control, stabilization of hypoalgesic priors, and reduced sensory gain of nociceptive input. However, these bands are context-dependent and not universal biomarkers. The alpha2 pattern suggests that, under hypnosis, subjective involuntariness may mediate the relationship between alpha activity and pain reduction, consistent with the role of agency-related processes in Hypnotic Hypoalgesia.	C/M-O	Moderate
6. Gamma reduction or gamma–pain decoupling during Hypnotic Hypoalgesia	Croft et al. (2002) [94]; De Pascalis et al. (2004) [95]	Gamma activity often correlates with pain intensity during waking or control conditions, but this relationship may weaken or disappear during Hypnotic Hypoalgesia. Some studies report reduced phase-ordered gamma responses under analgesic suggestions.	Gamma decoupling is consistent with reduced integration of nociceptive input into conscious pain experience. Within a predictive-coding interpretation, this may reflect reduced salience or altered sensory-evaluative integration, although it should not be taken as direct evidence that nociceptive prediction errors have been suppressed.	C/M-G	Moderate
7. Hyperalgesic suggestions increase pain-related salience markers	Arendt-Nielsen et al. (1990) [121]; De Pascalis et al. (2015) [120]; Ray et al. (2002) [119]; Valentini et al. (2013) [96]	Hyperalgesic suggestions can increase subjective pain or unpleasantness and may enhance P2/P250 and gamma responses, especially in high hypnotizable individuals. Some studies report subjective hyperalgesia without SERP change.	Hyperalgesia provides a reverse test of precision modulation: suggestions may increase pain when they enhance the precision of pain-related priors or increase the salience assigned to nociceptive input. Dissociated cases suggest that subjective inference can change without measured SERP amplitude changes.	C/M-L/G	Moderate
8. Autonomic and interoceptive modulation accompanying Hypnotic Hypoalgesia	De Pascalis & Perrone (1996) [157]; Houzé et al. (2021) [45]; Paqueron, et al. (2019) [158]; Rousseaux et al. (2022) [48]	Hypnotic Hypoalgesia may be accompanied by changes in autonomic markers, HRV, involuntariness, dissociation, or VR-induced absorption, suggesting that pain modulation involves interoceptive and agency-related processes as well as sensory processing.	These findings are consistent with an active-inference account of bodily state, in which suggestions may alter interoceptive priors, autonomic regulation, and the perceived agency of analgesic responses.	C/M-A/I	Moderate
9. Chronic pain and multiband network reorganization. Multiband cross-frequency coupling.	Jensen et al. (2018) [47]; Kumar Govindaiah et al. (2024) [83]	Clinical pain studies suggest that hypnosis and related interventions may alter theta, alpha, beta, and slow-gamma activity, although immediate pain reduction is not always observed. Chronic pain likely involves altered network-level dynamics rather than a single oscillatory abnormality. Theta–gamma, alpha–gamma, and alpha–beta interactions may be relevant, but direct evidence in Hypnotic Hypoalgesia remains limited.	Chronic pain may reflect maladaptive priors, persistent threat prediction, and altered interoceptive precision. Hypnosis may help recalibrate these maladaptive priors and precision assignments, but this interpretation remains preliminary and requires direct experimental testing. Cross-frequency coupling may provide a mechanistic bridge between top-down predictions and sensory-evaluative integration, but this remains a hypothesis requiring direct testing.	M-O/C	Low–Moderate

Tag legend: C = convergent evidence; D = dissociated evidence; M = mixed evidence; E = early sensory component; L = late evaluative component; O = oscillatory activity; G = gamma-related integration; A = agency/involuntariness; I = interoceptive/autonomic modulation. Confidence: High = pattern has been observed across multiple studies or is strongly supported by convergent ERP/EEG evidence. Moderate = pattern is plausible and supported by selected studies but remains conditional on hypnotizability, suggestion type, task, or analysis method. Low–Moderate = pattern is theoretically plausible and clinically relevant but currently supported by preliminary or indirect evidence. Interpretive caution: Predictive-coding and active-inference entries should be read as theoretically informed interpretations of the empirical findings, not as direct computational tests. In particular, gamma changes should not be treated as direct proof of prediction-error suppression, and cross-frequency coupling should be treated as a future analytic direction rather than an established mechanism of Hypnotic Hypoalgesia.

## Data Availability

The original contributions presented in this study are included in the article/Supplementary Materials. Further inquiries can be directed to the corresponding author.

## Supplementary Materials

The following supporting information can be downloaded at: https://www.mdpi.com/article/10.3390/brainsci16080779/s1, Figure S1: Simplified illustration of hierarchical predictive coding (PC) operations; Figure S2: Predictive coding model of hypnotic analgesia integrating bottom-up nociceptive signaling and top-down pain modulation. Section S1: Predictive Coding Framework (PCF); Section S2: Three PC Perspectives on Suggestion-Based Pain Modulation During Hypnosis.
