# Supplementary material for: Toward a Predictive Coding Account of Hypnotic Hypoalgesia: Integrating EEG Oscillations and Somatosensory Evoked Responses
Source: Brain Sci. 2026 Jul 23;16(8):779. doi: 10.3390/brainsci16080779 (PMC13510804; doi:10.3390/brainsci16080779)
Supplement: Supplementary file 1 [file brainsci-16-00779-s001.zip › brainsci-4419597-supplementary.pdf]

Supplementary Materials

Brain Sciences: Special Issue on "Hypnotherapy: from basic research to clinical practice."

Toward a Predictive Coding Account of Hypnotic Hypoalgesia: Integrating EEG Oscillations and Somatosensory Evoked Responses

Vilfredo De Pascalis [1,2]

[1] Department of Psychology, Sapienza University of Rome, 00185 Rome, Italy; vilfredo.depascalis@fondazione.uniroma1.it

[2] School of Psychology, University of New England, Armidale, NSW 2351, Australia

S1. Predictive Coding Framework (PCF)

In the latter half of the nineteenth century, von Helmholtz argued that the ambiguity of sensory input necessitates that perceptual systems rely on inferential processes [1]. Perception does not directly reflect the external world; it relies on prior knowledge and experience to resolve uncertainties in sensory input. The brain actively constructs plausible hypotheses about incoming sensory information [2]. According to a predictive coding (PC) perspective, the brain is an active player rather than a passive receiver of sensory inputs. It builds and tests models of the world while accounting for uncertainty [3].

Within a predictive coding framework (PCF), perception is described as an ongoing process of updating beliefs through three components: (i) "priors" existing expectations about sensory input; (ii) "sensory evidence"—incoming signals from the environment and the body; and (iii) "posteriors"—revised beliefs formed by integrating priors with new evidence. Empirically shaped priors generate top-down predictions via internal generative models, which are tested against afferent information from various sensory systems [3]. Thus, perception is an active, recursive process in which the brain uses prior knowledge to predict and refine its understanding of the world and the body.

In the context of "perceptual inference", when predictions do not align with the afferent data stream, the resulting discrepancy is termed prediction error (PE), i.e., the mismatch between the brain's top-down predictions (expectations about sensory input)

and the actual bottom-up sensory signals received from the environment (see Figure S1a) [4-6]. In the short term, PEs signal significant events and guide perceptual updates [3,7]. In the long term, persistent errors encourage learning by prompting the revision of prior predictions and updating their probabilities based on trial outcomes, thereby leading to more accurate predictions in future instances (see Figure S1b) [3].

Predictive coding (PC) is anchored in two main principles: hierarchical organization and precision weighting. It presents a robust framework for understanding how the brain shapes perception, cognition, and action by predicting the origins of sensory input [3,4].

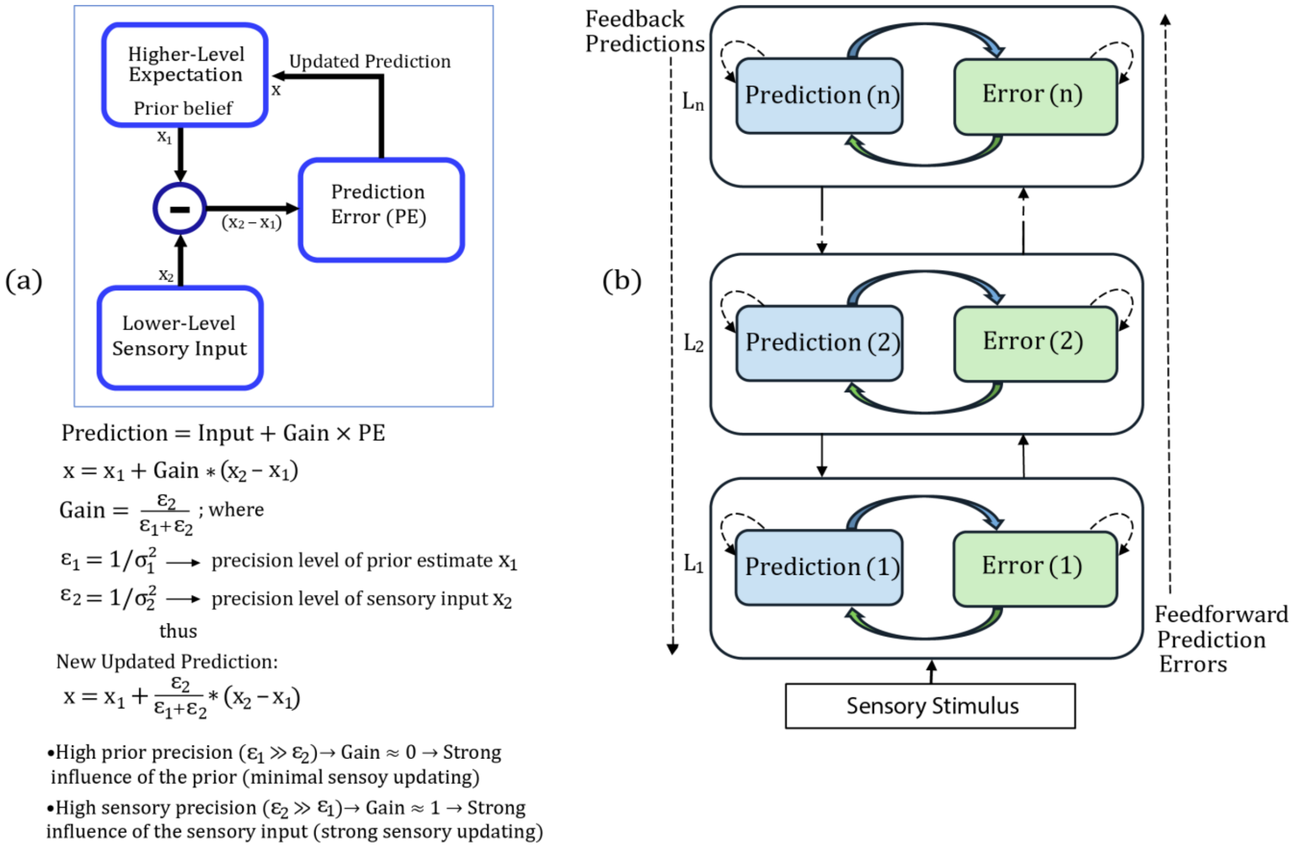

Figure S1. Simplified illustration of hierarchical predictive coding (PC) operations.
The figure in panel (a) essentially presents a Bayesian precision-weighted update rule, illustrating a local neural circuit that integrates bottom-up sensory inputs (e.g., nociceptive or other sensory signals) with top-down predictions from an internal generative model. PEs arise from mismatches between sensory input and top-down expectation (prior belief) and are used to update the model, thereby refining future predictions. In the bottom panel**,** a mathematically simplified single-level hierarchical PC computation illustrating how the current estimate x is updated through precision-weighted integration of bottom-up sensory input x_2 and top-down predictions x_1, wherein the PE represents a "surprise" signal, and the gain is determined as a function of the relative precision of x_1 and x_2.

Panel (b) represents multilayer PC models that are organized hierarchically. Signals ascend from lower sensory levels and convey PEs, whereas higher cortical levels send descending predictions related to meaning, self-referential expectations, and control.
[The Figure S1 above is an adapted version of panel A in Figure 2 of the open-access article by Z.S. Chen 2023 Chen [8]. Copyright © 2023 Chen; distributed under the terms of the Creative Commons Attribution License (CC BY); canonical URL https://creativecommons.org/licenses/by/4.0/].

In this framework, information processing occurs across multiple levels. Higher levels represent abstract information, such as prior knowledge and expectations, which generate predictions about sensory input. For example, based on context and experience, the system may anticipate a specific sound or object. This prediction guides the interpretation of incoming signals.

Lower-level processes handle immediate sensory information and compare it to predictions made by higher-level cognitive functions. When the actual sensory input aligns with these predictions, minimal updates are necessary. However, if a mismatch occurs, it is recorded as a "prediction error" and sent up to higher levels for adjustments to the predictions or beliefs [4,9]. Higher cognitive levels encode more stable beliefs and models, while lower levels quickly adapt predictions in response to changing sensory conditions [3,10,11]. Therefore, perception and action arise from the interaction between these stable higher-order models and the flexible processing of lower levels.

Because predictive systems operate under conditions of uncertainty, they must estimate not only what is being predicted and how much incoming input differs from that prediction, but also how reliable each source of information is. PC addresses this problem through precision weighting. Precision refers to the estimated reliability, confidence, or expected informativeness of a signal. In this context, precision weighting means that prediction errors are not treated equally: the system assigns greater weight to reliable errors and less weight to unreliable or noisy errors [9].

Precision, therefore, determines how strongly a prediction error influences belief updating. When a prediction error is assigned high precision, the system treats the discrepancy as meaningful evidence that the current model may need to be revised. Such errors are therefore amplified in their impact on subsequent processing. By contrast, when a prediction error is assigned low precision, the system treats the discrepancy as likely to reflect noise, ambiguity, or unreliable sensory input. These errors are therefore down-weighted and have little effect on updating higher-level predictions or beliefs [12].

PC also provides an efficiency rationale for perception. Since the brain lacks direct access to the external causes of sensory signals, it must infer these causes from changes in internal states [3]. Processing all raw sensory data in intricate detail would be both metabolically and computationally impractical. Instead, PC offers an economical strategy: predicting the next sensory state and encoding only what deviates from that prediction [4,13,14]. "Surprise" (i.e., prediction error) can occur at multiple hierarchical

levels [3,7]; a minor mismatch results in low-level visual sensory error. In contrast, a substantial mismatch (e.g., the unexpected presence of red shoes on a table rather than on a shelf) results in a higher-level error. Encoding these deviations rather than processing the entire signal is computationally efficient [3].

A broader theoretical claim in PCF is that organisms must minimize the long-run average of surprise, often framed as minimizing entropy, because otherwise they cannot maintain essential physiological variables within viable bounds [3,15,16]. Surprise can be reduced in two principal ways, depending on the agent's goals: either through perceptual inference or through "active inference". In perceptual inference, Bayesian belief updating is driven by weighted prediction errors (PEs); that is, when sensory evidence is sufficiently precise, the model revises previous expectations, whereas highly weighted expectations suppress updating and leave the generative model essentially unchanged [3,15,16] (see Fig. 1a). Precision weighting provides the mechanism by increasing the gain/precision of PEs, e.g., via selective attention to the perceived object, to boost their influence on updating [12]. Physiologically, this "gain" can be understood as postsynaptic excitability that governs the transmission of neuronal messages between prediction and error populations (see the mathematical definition of gain at the bottom of Fig. 1a). Psychologically, increased precision typically corresponds to selective attention. In contrast, decreased precision corresponds to "sensory attenuation" [17]. Without any sensory feedback, the agent focuses on minimizing the complexity of the generative model by reducing the difference between posterior and prior beliefs [16].

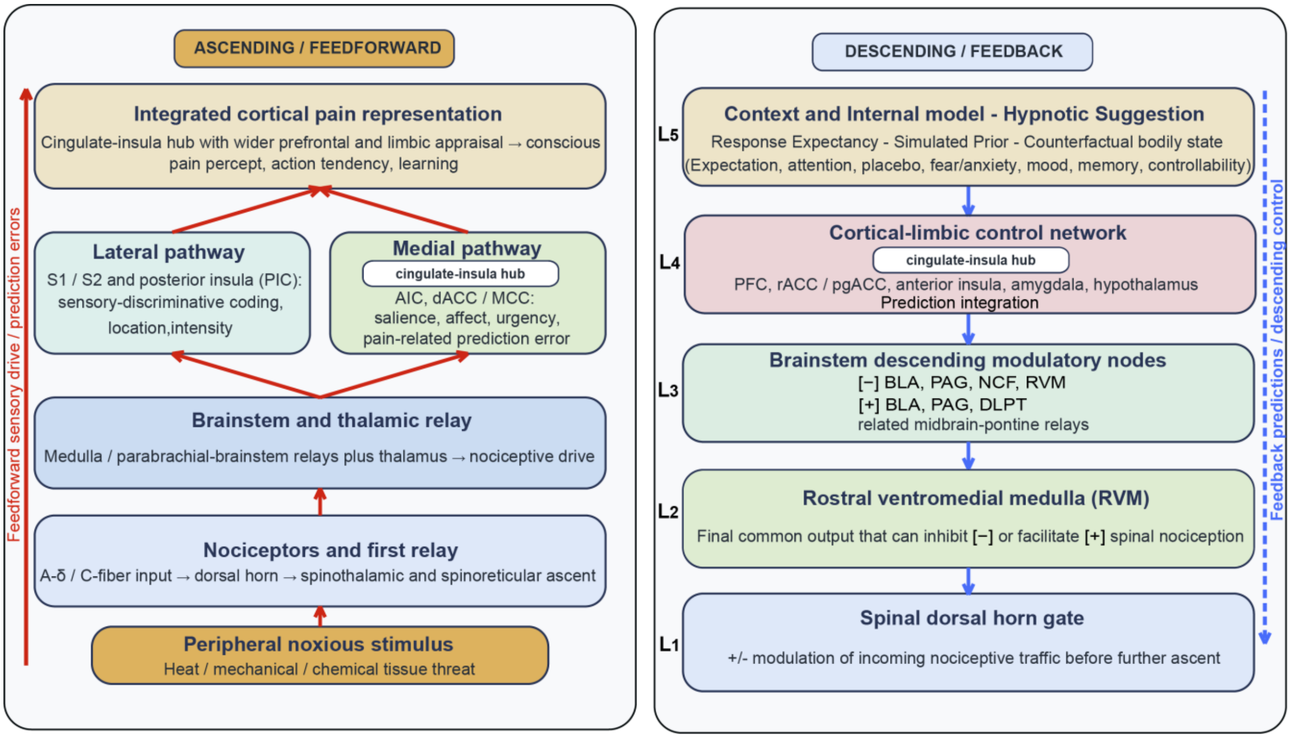

Figure S2. Predictive coding model of hypnotic analgesia integrating bottom-up nociceptive signaling and top-down pain modulation. Bottom-up (left panel) ascending sensory input conveys nociceptive prediction errors from the spinal cord and brainstem, including the rostral ventromedial medulla and PAG, to higher-order cortical systems involved in pain processing. At the cortical level, the cingulate–insula hub, comprising mainly the AIC and the ACC/AMCC, is proposed to integrate interoceptive and salience-related information with attentional and control processes. Upper cortical and subcortical structures generate top-down (right panel) predictions and modulatory influences that act on descending pain-control pathways, thereby shaping the precision and impact of ascending nociceptive prediction errors. In hypnosis, analgesic suggestions are hypothesized to alter these hierarchical inferential processes, reducing the conscious impact of nociceptive input through enhanced top-down control and altered precision weighting of sensory prediction errors.

Abbreviations: S1/S2, primary/secondary somatosensory cortices; PIC, posterior insula cortex; AIC, anterior insula cortex; ACC, anterior cingulate cortex; rACC, rostral anterior cingulate cortex; pgACC, pregenual anterior cingulate cortex; dACC, dorsal anterior cingulate cortex; MCC, middle cingulate cortex; AMCC, anterior midcingulate cortex; PCC, posterior cingulate cortex; PFC, prefrontal cortex; SMA, supplementary motor area; BLA, basolateral amygdala; PAG, periaqueductal gray; RVM, rostral ventromedial medulla; NCF, nucleus cuneiformis; DLPT, dorsolateral pontine tegmentum; PHG, parahippocampal gyrus;

The following abbreviations are used in this manuscript

Active inference is a theoretical framework that views perception and action as inference-based processes [18]. In this framework, actions minimize prediction errors (PEs) (see Figure S2) and share parallels with models like control-as-inference and belief propagation [19-21]. It is proposed that active inference is a generalized form of predictive coding that may explain both acute and chronic pain, with the cingulate–insula hub playing an important integrative role.

In "active inference", the neural system reduces prediction error not by changing beliefs but by changing the world, or the body, to make sensory evidence conform to predictions [22,23]. If an agent intends to grasp an object, it generates proprioceptive and somatosensory predictions about the hand's movement. In active inference, prediction error is attenuated not mainly by revising higher-level beliefs, but by acting on the body when motor predictions are assigned high precision. Under these conditions, top-down predictions dominate over incoming sensory evidence. Reflex arcs and peripheral motor mechanisms are essential for aligning sensory feedback with predicted movements [9,22]. Proprioceptive prediction errors primarily trigger neuromuscular reflexes to achieve the expected bodily state, rather than changing motor beliefs [22]. Thus, descending predictions represent prior intentions in the periphery, often leading to an "attenuation of ascending PEs," a hallmark of self-generated actions [23]. In other words, to move is, in a precise sense, to reduce the impact of sensory evidence that one is not moving. Significantly, despite conceptual differences, perceptual and active inference both minimize long-term surprise via reciprocal top-down and bottom-up prediction–error exchanges [23].

In acute pain, this model interprets nociceptive signals as clear threats, triggering immediate protective behavior responses or withdrawal responses to restore balance. These actions are essential for reducing uncertainty and aligning sensory input with the brain's predictions, minimizing prediction error [8].

In contrast, chronic pain arises from persistent prediction errors that cause the brain to inaccurately update its pain model, leading to the perception of threats without actual pain signals. This inaccurate update creates a cycle of false sensations, and reliance on flawed pain beliefs exacerbates the problem. The cingulate-insula network is key in maintaining these maladaptive beliefs by predicting bodily states and their emotional significance [8].

In sum, active inference provides a unified account in which both acute and chronic pain are outcomes of the same computational principle—minimization of prediction error—but differ in how effectively the brain updates its generative model in response to changing sensory evidence [18,19,21].

S2. Three PC Perspectives on Suggestion-Based Pain Modulation During Hypnosis

Here are briefly described three recent PC accounts of how hypnosis can modulate pain perception. PC models explain how hypnotic suggestions can modulate pain perception, suggesting that hypnotic suggestions of hypoalgesia involve a top-down reorganization of perceptual inference rather than a passive reduction of nociceptive input.

Here are briefly described three recent PC complementary accounts of how hypnosis can modulate pain perception.

(1) Simulation–Adaptation Theory of Hypnosis (SATH) suggests that hypnotic analgesia occurs through sustained cognitive simulation and focused attention on pain representations. This focus leads to an adaptive downregulation of the precision of pain-related prediction errors [24]

(2) Jamieson [25-27] has advanced a further predictive-coding interpretation of hypnosis, placing particular emphasis on the role of accepted response expectancies in organizing hypnotic experience. According to this view, hypnotic suggestions become more effective when they are incorporated into higher-order predictions, i.e., expectations about bodily states, and interact with interoceptive predictive processing and active inference. "Expectation" encompasses not just mental anticipation but also a predictive framework that shapes our conscious experiences and bodily functions. Accepted expectations can improve perception of internal signals, such as pain, heat, and tension. This alignment helps our body's regulatory systems, enhancing our overall well-being and comfort.

(3) A further prospect on hypnotic phenomena is provided by Martin and Pacherie's agency-based framework, which suggests that hypnotic suggestion affects how individuals perceive ownership and monitoring of sensation-related experiences [28]. From this viewpoint, changes in pain perception may stem from shifts in one's sense of

agency over pain. Consequently, hypnotic suggestions can alter both the intensity of pain and the interpretation of that pain as "mine," controllable, or beyond voluntary control.

S2.1. SATH and Hypnotic Pain Reduction

This proactive approach to pain relief is both effective and empowering. Deliberate use of vivid sensory imagery, including forms of sensory-obstructive imagery, can significantly alter an individual's experience of pain, highlighting how attention and imagination influence pain processing [29]. Within the SATH framework, Zahedi and colleagues [24] propose that perception relies not only on sensory signals but also on active, model-based representational inference, in which internal predictions shape our experiences.

When individuals mentally rehearse tasks and their responses, they enhance their cognitive abilities on various levels. This process helps solidify both straightforward stimulus-response connections and more complex ideas about rules, goals, and strategies. By viewing cognitive control through this lens, we can appreciate how context-sensitive associations between stimuli and responses flourish, influenced by rewards and challenges in our learning environments.

In the SATH framework, improvements in cognitive control involve not only proactive responses to pain but also anticipatory mechanisms that prepare individuals for task-relevant activities. Zahedi and Sommer [30] have highlighted that cognitive tasks require the generation of novel ideas and the use of flexible strategies, so that individuals receiving suggestions can benefit from visualizing tasks and creatively integrating those suggestions into their performance [31,32].

In the domain of pain regulation, sustained engagement with pain-related images or concepts—whether through focused attention, imagery, or deliberate transformation—may modify the neural systems involved in simulating, evaluating, and interpreting painful experience. Hypnotic hypoalgesia offers a clear example of this process. It may reduce neural networks' tendency to categorize incoming sensory signals as painful, allowing expectancy-based representations to exert greater influence on perception than nociceptive input alone.

This mechanism can be differentiated from placebo hypoalgesia. Placebo effects depend primarily on treatment-related contextual cues that elicit expectations and engage endogenous pain-modulatory systems [33]. Hypnotic hypoalgesia, by contrast, appears to involve the active construction of an alternative experiential state, one that changes not only expectations about pain but also the way pain is perceived, appraised, and regulated [24,34].

Consistent with this view Desmarteaux et al. [35] found that auditory suggestions for both hypoalgesia and hyperalgesia produced BOLD decreases in the parietal operculum and anterior midcingulate cortex (AMCC), and increases in the left parahippocampal gyrus (PHG), relative to a control condition. These suggestion-related

changes predicted subsequent pain-evoked responses: hyperalgesic suggestions were associated with increased activity in the ACC and AIC, whereas hypoalgesic suggestions were associated with reduced activity in the ACC, AMCC, posterior insula, and thalamus. These neural effects paralleled participants' subjective pain reports, indicating that hypnotic suggestions modulated both pain experience and brain systems engaged in its evaluation.

S2.2. Jamieson's Active Inference and Interoceptive PC Account

Jamieson's predictive processing approach expands the PC framework (PCF) by emphasizing the role of interoceptive predictive coding and active inference in hypnotic responding [25-27,36]. From this perspective, hypnotic responses emerge from active inferences—or response expectancies—embedded within hierarchical self–world models that become engaged when individuals accept hypnotic suggestions. Rather than functioning merely as instructions, hypnotic suggestions encourage the adoption of counterfactual generative models. For example, when a painful stimulus is applied to the left hand, a participant might be encouraged to adopt beliefs such as "the stimulus is harmless" or "my hand feels numb, as if a plastic glove protected it." Once these suggestion-based beliefs are accepted, they guide active inference processes that reduce prediction errors by modifying perception, action, and bodily experience. Jamieson argues that these effects rely heavily on interoceptive predictive coding, particularly by suppressing interoceptive prediction errors. Consequently, activity within salience and executive control networks decreases, leading to a dissociated mode of control.

Within this framework, information processing is organized hierarchically across different regions of the insula (see Figure S2, right-quadrant). At the lowest level, the posterior insula encodes primary interoceptive and somatosensory prediction errors related to pain and sensorimotor functions [37,38]. Above this, the dorsal AIC integrates interoceptive information with demands related to attention, salience, and cognitive control [39,40]. The ventral AIC integrates predictions about bodily states with emotional and self-referential representations that are important for emotional and social processing [41-43]. This view, centered on the insula, aligns with Chen's suggestion of a cingulate–insula hub that coordinates predictive coding of pain [8]. Additionally, event-related potential (ERP) studies have shown that hypnotic hypoalgesia reduces both early sensory and later evaluative components of sensory responses.

From the perspective of active inference, hypnotic hypoalgesia involves more than a decrease in sensory precision; it entails a self-reinforcing cycle in which perception and action align with suggested bodily states [27,44]. Current theories of hypnosis, as discussed by Jamieson and Woody [45] and Woody and Sadler [46], suggest that hypnotic responses result from the disengagement of the central executive network or weakened connectivity with the salience network. Embracing and experiencing hypnotic

suggestions involves fascinating interoceptive predictive coding mechanisms similar to those found in virtual reality.

More specifically, top-down interoceptive predictions are compared with bottom-up bodily signals within the ventral AIC. When interoceptive prediction errors remain minimal, interoceptive prediction units become temporarily stabilized, and the dorsal AIC does not recruit attentional or executive control systems. Bodily feedback can update generative models through prediction-error correction. However, active inference also allows existing generative models—such as hypnotically induced response expectancies—to function as physiological set points that shape autonomic reflexes, including the baroreflex. This mechanism, termed *interoceptive inference*, was originally proposed by Seth and colleagues [47,48] and by Gu et al. [49].

Jamieson [26] emphasizes the fascinating role of interoceptive inference in shaping the strong sense of reality associated with hypnosis. Immediately following the suggestions of hypnotic hypoalgesia, expectations about bodily states are thought to minimize incoming nociceptive and interoceptive prediction errors. In other words, the brain's predictions about reduced pain begin to outweigh or suppress sensory signals that would normally indicate discomfort.

Expectation-driven processes aim to decrease activity in both the salience network and executive control systems. This reduction can help individuals feel less pain and experience a diminished sense of voluntary control. As a result, many people view their responses to hypnosis as occurring automatically rather than through intentional effort. In this context, hypnotic hypoalgesia demonstrates that the brain effectively manages bodily sensations through prediction-based mechanisms rather than by altering attention or sensory reinterpretation.

From this perspective, hypnotic hypoalgesia serves as an intriguing example of active inference. In this process, the brain actively influences our bodily experiences through predictive mechanisms, extending beyond simple shifts in attention or mere reinterpretation of sensory perceptions.

S2.3. Martin and Pacherie's agency-based account

Martin and Pacherie present an engaging agency-based perspective on hypnotic phenomena that is quite captivating. Instead of concentrating on the brain's predictive or precision-weighting mechanisms, they explore how our sense of agency can shift during hypnosis. This perspective aligns well with the cold control theory [50-52], which suggests that hypnotic responses arise from significant changes in metacognitive monitoring and agency attribution, rather than a complete loss of intention.

According to Martin & Pacherie's agency-based account [28], hypnotic hypoalgesia offers a fascinating glimpse into how our minds process pain.

The agency-based account suggests that, while our responses to pain still occur through intact control systems, they go unrecognized by our higher-order monitoring

system, leading to a reduced sense of ownership over those sensations. This shift transforms our experience of pain through hypnotic suggestion, demonstrating that it is not merely about dulling sensory input—it is a remarkable reconfiguration of our perception of agency.

S2.4. A comparative perspective account

The three predictive models of hypnotic modulation we explored challenge the idea that hypnotic hypoalgesia is merely a peripheral or reflexive response. Instead, they emphasize that pain reduction during hypnosis results from a complex interplay of top-down predictive processes operating at multiple, interconnected levels of cognition and neural organization. While all three models acknowledge the impact of hypnosis on PC processing, they each highlight different aspects that contribute to its remarkable analgesic effects.

From the perspective of advancing hypnosis-based pain treatment, each approach contributes a distinct theoretical insight.

The SATH framework [24] contributes a detailed account of attentional dynamics and precision modulation. By emphasizing representational simulation and neural adaptation, SATH explains how hypnotic suggestions stabilize alternative perceptual interpretations and reduce the salience of nociceptive signals. Its contribution lies in clarifying how sustained cognitive focus and altered precision weighting can weaken pain processing without necessarily relying on overt behavioral regulation. In clinical contexts, this perspective helps explain how hypnotic techniques may selectively reduce the impact of pain signals while preserving broader cognitive functioning.

Jamieson [26], in his interoceptive predictive coding and active inference account, highlights the role of bodily inference and descending regulation, offering a mechanistic explanation for how hypnotic suggestions can alter the subjective experience of pain by reshaping interoceptive expectations and minimizing prediction error. This framework is particularly important for linking hypnotic analgesia to broader models of brain–body regulation and for explaining how cognitive expectations can produce measurable physiological and experiential changes.

Martin and Pacherie [28] enhance our understanding of hypnotic analgesia by highlighting the essential aspects of agency, authorship, and metacognition. Their innovative model provides insight into the intriguing phenomenon of automatic pain reduction during hypnosis. By examining how altered access to intentions and self-monitoring improves the effectiveness of hypnotic suggestions, they clarify why these processes can feel effortless and compelling. This significant contribution enhances our understanding of patient responsiveness and compliance and sheds light on the unique experiences arising from effective hypnotic interventions, ultimately advancing clinical applications in pain management.

Taken together, these approaches significantly broaden the conceptual foundation of hypnosis-based pain treatment. Rather than offering competing explanations, they describe complementary dimensions of the same hierarchical predictive system.
These three models, when considered together, advance our understanding of hypnotic hypoalgesia by integrating neurophysiological, cognitive, and phenomenological aspects into a multidimensional framework. Importantly, this convergence has significant implications for both clinical practice and future research. The three PC approaches suggest that effective hypnotic analgesia relies on coordinated changes across perceptual, interoceptive, and metacognitive systems.

This integrated perspective moves hypnosis research beyond previous debates about the "real" or "merely suggestive" nature of the hypnotic suggestion response. Instead, the hypnotic hypoalgesia response is interpreted as a complex form of predictive regulation that alters the conscious experience of pain through interconnected neural mechanisms of precision weighting, bodily inference, and agency attribution.

References

1. von Helmholtz, H. Concerning the perceptions in general. In *Visual Perception: Essential Readings*, Yantis, S., Ed. Taylor & Francis: Philadelphia, 2001; pp. 24-44.
2. Gregory, R.L. Knowledge in perception and illusion. *Philosophical Transactions of the Royal Society B: Biological Sciences* **1997**, *352*, 1121-1127, doi:10.1098/rstb.1997.0095.
3. Clark, A. Whatever next? Predictive brains, situated agents, and the future of cognitive science. *Behavioral and Brain Sciences* **2013**, *36*, 181-204, doi:10.1017/S0140525X12000477.
4. Friston, K. The free-energy principle: a unified brain theory? *Nature Reviews Neuroscience* **2010**, *11*, 127-138, doi:10.1038/nrn2787.
5. Friston, K. A theory of cortical responses. *Philosophical Transactions of the Royal Society B: Biological Sciences* **2005**, *360*, 815-836, doi:10.1098/rstb.2005.1622.
6. Rao, R.P.N.; Ballard, D.H. Predictive coding in the visual cortex: a functional interpretation of some extra-classical receptive-field effects. *Nature Neuroscience* **1999**, *2*, 79-87, doi:10.1038/4580.
7. Barron, H.C.; Auksztulewicz, R.; Friston, K. Prediction and memory: A predictive coding account. *Progress in Neurobiology* **2020**, *192*, 101821, doi:10.1016/j.pneurobio.2020.101821.
8. Chen, Z.S. Hierarchical predictive coding in distributed pain circuits. *Frontiers in Neural Circuits* **2023**, *Volume 17 - 2023*, doi:10.3389/fncir.2023.1073537.

9. Yon, D.; de Lange, F.P.; Press, C. The Predictive Brain as a Stubborn Scientist. *Trends in Cognitive Sciences* **2019**, *23*, 6-8, doi:10.1016/j.tics.2018.10.003.
10. Jones, M.; Wilkinson, S.; Abraham, A. From prediction to imagination. *The Cambridge handbook of the imagination* **2020**, 10.1017/9781108580298.007, 94-110, doi:10.1017/9781108580298.007.
11. Beni, M.D. A tale of two architectures: Free energy, its models, and modularity. *Consciousness and Cognition* **2022**, *98*, 103257, doi:10.1016/j.concog.2021.103257.
12. Auksztulewicz, R.; Friston, K. Attentional Enhancement of Auditory Mismatch Responses: a DCM/MEG Study. *Cerebral Cortex* **2015**, *25*, 4273-4283, doi:10.1093/cercor/bhu323.
13. Friston, K. Prediction, perception and agency. *International Journal of Psychophysiology* **2012**, *83*, 248-252, doi:https://doi.org/10.1016/j.ijpsycho.2011.11.014.
14. Friston, K.; Price, C.J. Dynamic representations and generative models of brain function. *Brain Research Bulletin* **2001**, *54*, 275-285, doi:10.1016/S0361-9230(00)00436-6.
15. Friston, K.; Schwartenbeck, P.; Fitzgerald, T.; Moutoussis, M.; Behrens, T.; Dolan, R.J. The anatomy of choice: active inference and agency. *Frontiers in Human Neuroscience* **2013**, *Volume 7 - 2013*, doi:10.3389/fnhum.2013.00598.
16. Friston, K.; Wiese, W.; Hobson, J.A. Sentience and the Origins of Consciousness: From Cartesian Duality to Markovian Monism. In *Entropy*, 2020; Vol. 22, p 516.
17. Hohwy, J. Attention and Conscious Perception in the Hypothesis Testing Brain. *Frontiers in Psychology* **2012**, *Volume 3 - 2012*, doi:10.3389/fpsyg.2012.00096.
18. Pezzulo, G.; Rigoli, F.; Friston, K. Active Inference, homeostatic regulation and adaptive behavioural control. *Progress in Neurobiology* **2015**, *134*, 17-35, doi:10.1016/j.pneurobio.2015.09.001.
19. Friston, K.; Parr, T.; de Vries, B. The graphical brain: Belief propagation and active inference. *Network Neuroscience* **2017**, *1*, 381-414, doi:10.1162/NETN_a_00018.
20. Millidge, B.; Tschantz, A.; Seth, A.K.; Buckley, C.L. On the Relationship Between Active Inference and Control as Inference. In Proceedings of Active Inference, Cham, 2020//; pp. 3-11.
21. Seymour, B.; Mancini, F. Hierarchical models of pain: Inference, information-seeking, and adaptive control. *NeuroImage* **2020**, *222*, 117212, doi:10.1016/j.neuroimage.2020.117212.
22. Adams, R.A.; Shipp, S.; Friston, K. Predictions not commands: active inference in the motor system. *Brain Structure and Function* **2013**, *218*, 611-643, doi:10.1007/s00429-012-0475-5.
23. Brown, H.; Adams, R.A.; Parees, I.; Edwards, M.; Friston, K. Active inference, sensory attenuation and illusions. *Cognitive Processing* **2013**, *14*, 411-427, doi:10.1007/s10339-013-0571-3.

24. Zahedi, A.; Lynn, S.J.; Sommer, W. Cognitive simulation along with neural adaptation explain effects of suggestions: a novel theoretical framework. *Frontiers in Psychology* **2024**, *Volume 15 - 2024*, doi:10.3389/fpsyg.2024.1388347.
25. Jamieson, G.A. A unified theory of hypnosis and meditation states: The interoceptive predictive coding approach. In *Hypnosis and Meditation: Towards an Integrative Science of Conscious Planes*, Raz, A., Lifshitz, M. , Ed. Oxford University Press: New York, NY, 2016; pp. 313-342.
26. Jamieson, G.A. Expectancies of the future in hypnotic suggestions. *Psychology of Consciousness: Theory, Research, and Practice* **2018**, *5*, 258, doi:10.1037/cns0000170.
27. Jamieson, G.A. An insula view of predictive processing in hypnotic responses. *Psychology of Consciousness: Theory, Research, and Practice* **2022**, *9*, 117-129, doi:10.1037/cns0000266.
28. Martin, J.-R.; Pacherie, E. Alterations of agency in hypnosis: A new predictive coding model. *Psychological review* **2019**, *126*, 133, doi:10.1037/rev0000134.
29. Zahedi, A.; Lynn, S.J.; Sommer, W. How hypnotic suggestions work–A systematic review of prominent theories of hypnosis. *Consciousness and cognition* **2024**, *123*, 103730, doi:10.1016/j.concog.2024.103730.
30. Zahedi, A.; Sommer, W. The effects of task-relevant suggestions on motivated cognitive control. . In *International Review of Neurobiology: Hypnosis Part B*, D. B. Terhune, G.A.J., & V. De Pascalis (Eds.), Ed. Academic Press: New York, 2025; Vol. 185, pp. 1-30.
31. Zahedi, A.; Stuermer, B.; Hatami, J.; Rostami, R.; Sommer, W. Eliminating stroop effects with post-hypnotic instructions: Brain mechanisms inferred from EEG. *Neuropsychologia* **2017**, *96*, 70-77, doi:10.1016/j.neuropsychologia.2017.01.006.
32. Zahedi, A.; Stürmer, B.; Sommer, W. Can posthypnotic suggestions boost updating in working memory? Behavioral and ERP evidence. *Neuropsychologia* **2020**, *148*, 107632, doi:10.1016/j.neuropsychologia.2020.107632.
33. Büchel, C.; Geuter, S.; Sprenger, C.; Eippert, F. Placebo Analgesia: A Predictive Coding Perspective. *Neuron* **2014**, *81*, 1223-1239, doi:10.1016/j.neuron.2014.02.042.
34. Kirsch, I. Chapter Five - Response Expectancy and the Placebo Effect. In *International Review of Neurobiology*, Colloca, L., Ed. Academic Press: 2018; Vol. 138, pp. 81-93.
35. Desmarteaux, C.; Streff, A.; Chen, J.-I.; Houzé, B.; Piché, M.; Rainville, P. Brain Responses to Hypnotic Verbal Suggestions Predict Pain Modulation. *Frontiers in Pain Research* **2021**, *Volume 2 - 2021*, doi:10.3389/fpain.2021.757384.
36. Jamieson, G.A. Interoceptive predictive coding, active inference and the generation of hypnotic suggestion responses. In *International Review of Neurobiology: Hypnosis Part B*, Terhune, D.B., Jamieson, G.A., De Pascalis, V., Eds. Academic Press: New York, 2025; Vol. 185, pp. 31-57.

37. Craig, A.D. How do you feel? Interoception: the sense of the physiological condition of the body. *Nature Reviews Neuroscience* **2002**, *3*, 655-666, doi:10.1038/nrn894.
38. Wager, T.D.; Rilling, J.K.; Smith, E.E.; Sokolik, A.; Casey, K.L.; Davidson, R.J.; Kosslyn, S.M.; Rose, R.M.; Cohen, J.D. Placebo-induced changes in FMRI in the anticipation and experience of pain. *Science* **2004**, *303*, 1162-1167.
39. Dosenbach, N.U.F.; Visscher, K.M.; Palmer, E.D.; Miezin, F.M.; Wenger, K.K.; Kang, H.C.; Burgund, E.D.; Grimes, A.L.; Schlaggar, B.L.; Petersen, S.E. A Core System for the Implementation of Task Sets. *Neuron* **2006**, *50*, 799-812, doi:10.1016/j.neuron.2006.04.031.
40. Eckert, M.A.; Menon, V.; Walczak, A.; Ahlstrom, J.; Denslow, S.; Horwitz, A.; Dubno, J.R. At the heart of the ventral attention system: The right anterior insula. *Human Brain Mapping* **2009**, *30*, 2530-2541, doi:10.1002/hbm.20688.
41. Sanfey, A.G.; Rilling, J.K.; Aronson, J.A.; Nystrom, L.E.; Cohen, J.D. The Neural Basis of Economic Decision-Making in the Ultimatum Game. *Science* **2003**, *300*, 1755-1758, doi:doi:10.1126/science.1082976.
42. Chang, L.J.; Yarkoni, T.; Khaw, M.W.; Sanfey, A.G. Decoding the Role of the Insula in Human Cognition: Functional Parcellation and Large-Scale Reverse Inference. *Cerebral Cortex* **2013**, *23*, 739-749, doi:10.1093/cercor/bhs065.
43. Chang, Luke J.; Smith, A.; Dufwenberg, M.; Sanfey, Alan G. Triangulating the Neural, Psychological, and Economic Bases of Guilt Aversion. *Neuron* **2011**, *70*, 560-572, doi:10.1016/j.neuron.2011.02.056.
44. Blakemore, S.J.; Oakley, D.A.; Frith, C.D. Delusions of alien control in the normal brain. *Neuropsychologia* **2003**, *41*, 1058-1067, doi:https://doi.org/10.1016/S0028-3932(02)00313-5.
45. Jamieson, G.A.; Woody, E. Dissociated control as a paradigm for cognitive neuroscience research and theorizing in hypnosis. In *Hypnosis and conscious states: The cognitive neuroscience perspective*, Jamieson, G.A., Ed. Oxford University press: NY, 2007; pp. 111-129.
46. Woody, E.Z.; Sadler, P. Dissociation theories of hypnosis. In *The Oxford Handbook of Hypnosis: Theory, Research, and Practice*, A, N.M.a.B., Ed. Oxford University Press Inc.: New York, 2008; pp. 81-110.
47. Seth, A.K. Interoceptive inference, emotion, and the embodied self. *Trends in Cognitive Sciences* **2013**, *17*, 565-573, doi:10.1016/j.tics.2013.09.007.
48. Seth, A.K.; Friston, K. Active interoceptive inference and the emotional brain. *Philosophical Transactions of the Royal Society B: Biological Sciences* **2016**, *371*, 20160007, doi:10.1098/rstb.2016.0007.
49. Gu, X.; Hof, P.R.; Friston, K.; Fan, J. Anterior insular cortex and emotional awareness. *Journal of Comparative Neurology* **2013**, *521*, 3371-3388, doi:10.1002/cne.23368.

50. Dienes, Z.; Hutton, S. Understanding hypnosis metacognitively: rTMS applied to left DLPFC increases hypnotic suggestibility. *Cortex* **2013**, *49*, 386-392, doi:10.1016/j.cortex.2012.07.009.
51. Dienes, Z.; Perner, J. Executive control without conscious awareness: The cold control theory of hypnosis. In *Hypnosis and conscious states: The cognitive neuroscience perspective*, Jamieson, G.A., Ed. Oxford University Press: NY, 2007; pp. 293-314.
52. Dienes, Z.; Beran, M.; Brandl, J.L.; Perner, J.; Proust, J. Is hypnotic responding the strategic relinquishment of metacognition. *Foundations of metacognition* **2012**, 267-277.
